# Functional interaction between receptor tyrosine kinase MET and ETS transcription factors promotes prostate cancer progression

**DOI:** 10.1002/1878-0261.13739

**Published:** 2024-10-07

**Authors:** Elisa Carouge, Clémence Burnichon, Martin Figeac, Shéhérazade Sebda, Nathalie Vanpouille, Audrey Vinchent, Marie‐José Truong, Martine Duterque‐Coquillaud, David Tulasne, Anne Chotteau‐Lelièvre

**Affiliations:** ^1^ UMR9020 – UMR1277 – Canther – Cancer Heterogeneity, Plasticity and Resistance to Therapies Institut Pasteur de Lille, Univ. Lille, CNRS, Inserm, CHU Lille France; ^2^ US 41 – UAR 2014 – PLBS Institut Pasteur de Lille, Univ. Lille, CNRS, Inserm, CHU Lille France

**Keywords:** Capmatinib, ETS transcription factors, MET signalling, prostate cancer, transcriptomic analysis

## Abstract

Prostate cancer, the most common malignancy in men, has a relatively favourable prognosis. However, when it spreads to the bone, the survival rate drops dramatically. The development of bone metastases leaves patients with aggressive prostate cancer, the leading cause of death in men. Moreover, bone metastases are incurable and very painful. Hepatocyte growth factor receptor (MET) and fusion of genes encoding E26 transformation‐specific (ETS) transcription factors are both involved in the progression of the disease. ETS gene fusions, in particular, have the ability to induce the migratory and invasive properties of prostate cancer cells, whereas MET receptor, through its signalling cascades, is able to activate transcription factor expression. MET signalling and ETS gene fusions are intimately linked to high‐grade prostate cancer. However, the collaboration of these factors in prostate cancer progression has not yet been investigated. Here, we show, using cell models of advanced prostate cancer, that ETS translocation variant 1 (ETV1) and transcriptional regulator ERG (ERG) transcription factors (members of the ETS family) promote tumour properties, and that activation of MET signalling enhances these effects. By using a specific MET tyrosine kinase inhibitor in a humanised hepatocyte growth factor (HGF) mouse model, we also establish that MET activity is required for ETV1/ERG‐mediated tumour growth. Finally, by performing a comparative transcriptomic analysis, we identify target genes that could play a relevant role in these cellular processes. Thus, our results demonstrate for the first time in prostate cancer models a functional interaction between ETS transcription factors (ETV1 and ERG) and MET signalling that confers more aggressive properties and highlight a molecular signature characteristic of this combined action.

## Graphical abstract

The MET receptor activation pathway is intrinsically linked to the activity of the transcription factors ETV1 and ERG (ETS family) to promote tumorigenesis in prostate cancer cells. Here we focus on the transcriptional programme leading to this partnership and have identified 5 commonly regulated target genes that may represent a potential signature of prostate cancer progression at advanced stages.

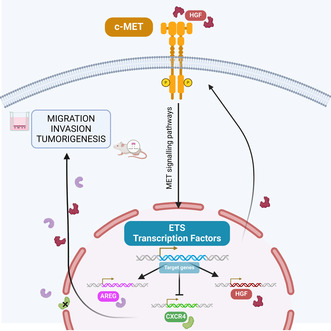

**Abbreviations**
AKT: protein kinase B
AREG: amphiregulin
BCL2: B‐cell lymphoma 2
BMP: bone morphogenetic protein
CXCR4: C‐X‐C chemokine receptor type 4
ELK1: ETS like‐1
ERG: ETS‐related gene
ETS: E‐twenty‐six (transcription factors)
ETV1: E‐twenty‐six variant 1
FBS: fetal bovine serum
HGF: hepatocyte growth factor
IL11: interleukin‐11
KITLG: kit ligand
MAPK: mitogen‐activated protein kinase
MMP: matrix metalloproteinase
MYC: myelocytomatosis oncogene
OSCAR: osteoclast‐associated receptor
PCa: prostate cancer
PI3K: phosphoinositide 3‐kinase
SLUG: zinc finger protein SNAI2
SNAIL: zinc finger protein SNAI1
TKI: tyrosine kinase inhibitor
TWIST: twist family BHLH transcription factor 1

## 1 Introduction

Prostate cancer (PCa) is one of the main malignant diseases affecting men worldwide, with a peak of 60% cases arising among men aged 65 and older [1, 2]. In France, while a 5‐year survival rate of approximately 93% brings hope [3], a subset of patients inevitably confronts to the therapy resistance, culminating in the insidious emergence of metastatic lesions within the bones, lymphatic nodes, and the liver [4, 5, 6]. Inevitably, metastatic infiltration of bone, a condition that occurs in 80% of cases, eludes existing curative interventions, invariably subjecting patients to pain that often ends in death [7].

Transcription factors belonging to the Erythroblast Transformation Specific (ETS) family are well‐established oncogenic factors implicated in physiological development as well as in a multitude of cancer types, including melanoma, Ewing's sarcoma, breast cancer and gastric cancer [8, 9, 10, 11, 12, 13]. These transcription factors exhibit the capacity to modulate target genes like epithelial‐mesenchymal transition genes such as *SNAIL*, *SLUG* and *TWIST1*, various matrix metalloproteinases (MMPs), cell cycle regulation as *CCND2* and apoptosis as *BCL2* genes, which orchestrate the malignant transformation of cancer cells and the development of metastatic lesions [14, 15, 16, 17, 18, 19].

In prostate cancer, ETS transcription factor‐encoding genes are retrieved as chromosomal rearrangements involving androgen‐dependent promoters. In 2005, Tomlins *et al*. underlined the pivotal roles of ERG (ETS‐related gene) and ETV1 (ETS translocation variant 1) in the landscape of prostate cancer [20]. Such rearrangements occur in 10% of PCa cases for ETV1 and 60% for ERG, invariably inducing excessive expression of ETV1 and ERG, thereby leading to the overexpression of their downstream targets [21]. These fusions trigger events encompassing acute cell proliferation, invasive potential and migratory capacity contributing to the aggressiveness of the malignant tumour [22, 23, 24, 25].

MET is a transmembrane receptor with tyrosine kinase activity, activated by the hepatocyte growth factor (HGF) and a recognised oncogene, whose amplification or mutation orchestrates events linked to the carcinogenic process and therapeutic relapse [26, 27, 28].

MET signalling acts by phosphorylating various kinases, such as MAPK or PI3K/AKT, thereby modulating the expression of target genes like *SLUG*, *MMP2* and *BMP2* involved in motility, invasion, the cell cycle survival and tumour progression [29, 30, 31, 32, 33].

MET has been widely documented as an important therapeutic target. Indeed, there is substantial evidence of its potential in clinical trials, particularly in the context of lung cancer, where MET‐targeted therapies have been shown to improve survival rates [34]. In addition, more than 40 clinical trials are currently underway investigating the use of Tyrosine Kinase Inhibitor (TKI) and particularly Capmatinib, a specific MET inhibitor, in the treatment of different cancers [35, 36, 37]. This underlines the growing interest and potential of therapies targeting MET in various malignant tumours.

ETV1 and ERG fusions are present throughout the progression of prostate cancer [38, 39, 40, 41], while MET, whose expression is repressed by the androgen receptor, appears mainly in advanced PCa and bone metastasis [42]. Interestingly, links between MET and ETV1/ERG have been uncovered, supporting their belonging to the same biological pathways. Indeed, some data has shown that ETS factors could be activated by MET pathways such as the MAPK signalling pathway [43, 44, 45, 46].

In this study, by using cellular models of advanced prostate cancer, we undertake an investigation of ETV1/ERG transcription factors and MET signalling interplay in tumour progression mechanic. We first depicted a regulation loop enabling MET and ETV1/ERG factors to regulate each other. We next demonstrated that these ETS factors as well as MET induced migratory capabilities and cell invasiveness and that these effects are implemented by their association. Moreover, we showed in a model of human HGF knock‐in mice that the pro‐tumour property of ETV1/ERG factors is closely linked to MET activity and proposed a specific molecular signature of this cooperation after conducting a large‐scale transcriptomic analysis. Thus, our results suggest that the collective influence of ETV1/ERG and MET signalling can have a significant impact on the trajectory of prostate cancer progression, precipitating its ascent to high‐grade malignancy.

## 2 Materials and methods

### 2.1 Cell culture, treatment and reagent

PC‐3M‐luc‐C6 (RRID:CVCL_D577), referred as PC3M in the article, and PC3 (RRID:CVCL_0035) human prostate cancer cells were originally purchased from Caliper Life Sciences© and the American Type Culture Collection© (ATCC) respectively. Cells are maintained in ATCC recommended medium and supplemented with 10% foetal bovine serum (DUTSCHER); 5% l‐glutamine (Gibco™, Waltham, MA, USA; REF: 25030081) as well as 5% sodium Pyruvate (Gibco™; REF: 11360070) when required. All cells were cultured at 37 °C and 5% CO_2_ in a humid atmosphere. Cells are passaged every 3–4 days to maintain them at approximately 80% confluence. HEK‐293T were purchased the American Type Culture Collection© (ATCC) and maintain in DMEM (Thermo Scientific™, Waltham, MA, USA, REF: 61965059) with 10% FBS and 5% l‐glutamine. Cells were used for retrovirus production. Cells are tested every month for potential mycoplasma contamination using the MycoAlert™ kit (Lonza^®^, Basel, Switzerland). During experiments, cells were treated with HGF concentrated at 20 ng⋅mL^−1^ and Capmatinib at 10 nm. We confirm that the cell lines have been authenticated within the last 3 years. Cell line authentication was performed by isolating DNA from a cell pellet. The genetic characteristics of the cell lines were then determined using PCR‐single‐locus technology. This method, developed by Eurofins Genomics, enables the specific genetic profiles of the cell lines to be detected accurately and reliably, guaranteeing their correct identification.

### 2.2 Production of viral vectors

Viral vectors for expression of ETV1 and ERG (10.18632/oncotarget.14399) transcription factors were constructed following amplification of the complementary DNA fragments by PCR. These complementary DNA fragments of ETV1 and ERG were then inserted into a retroviral plasmid vector plpcx puro using the In‐Fusion^®^ HD Cloning technique (Clontech, Saint Germain en Laye, France, REF: 102518). The various plasmids thus obtained were validated by sequencing and used to insert the various cell lines and PC3M and PC3 via retroviral infection.

### 2.3 Retroviral infection

The cells used for virus production were then transfected with the various plasmids required for retrovirus production (VSV‐G plasmid and plpcx Ctrl; plpcx ETV1 or plpcx ERG). The cells were transfected with corresponding plasmids each containing different sequences, named ETV1 and ERG. The HEK‐293 FT cells produced the retrovirus for 24 h then the viruses were harvested and deposited on the PC3M and PC3 cells. Infection was carried out for 24 h before changing the medium for one containing the antibiotic used to select infected cells. As the plpcx plasmid contains a puromycin resistance gene, cells that have integrated the virus will escape selection. Puromycin (Thermo Scientific™, REF: 1113803) was used at 1 μm concentration.

### 2.4 RNA extraction, reverse transcription and real‐time qPCR

Total RNA was extracted using the “NucleoSpin^®^ RNA” kit (Macherey Nagel, Hoerdt, France; REF: 740955) according to the manufacturer's instructions. Reverse transcription of total RNA (1 μg) was performed using the HighCapacity cDNA Reverse transcription Kit (Applied Biosystem^®^, Waltham, MA, USA; REF: 4368814). Expression of specific genes was determined by real‐time qPCR using the Power SYBR^®^ Green PCR Master Mix (Appliedbiosystem^®^; REF: 4367659) and the AriaMix system (AriaMx Real‐time PCR System; Agilent Technology^®^, les Ulis, France). Results were analysed using the comparative cycle threshold method normalised to the *TBP* housekeeping gene and compared to a comparator sample using the Delta–Delta Ct (2−^∆∆Ct^) method. The nucleotide sequences of the primers used are in supplementary data (Table S1).

### 2.5 Protein extraction and western blotting

Cells proteins were extracted using the PY buffer (SDS and Triton 10%) supplemented by phosphatase and protease inhibitors (PMSF [10%], Leupeptin [1%], NA_3_VO_4_ [1%], βGlycerophosphate [20%], Aprotinin [10%]), and separated in 4–12% precasted gel (NuPAGE™ 4 à 12%, Bis‐Tris). After migration, samples were transferred by electrophoresis to PVDF membrane. Membranes were incubating with blocking buffer during 30 min, then with primary antibody over the night. Membranes were incubated with secondary HRP‐conjugated antibody for 1 h. After washing, membranes were revealed using DURA™ Western ECL substrate (Bio‐Rad, Hercules, CA, USA). The antibodies used are Phospho‐MET (Cell Signalling, MA, USA, #3077), MET (Cell Signalling – #3148), Phospho‐AKT (Cell Signalling – #4060), AKT (Cell Signalling – #2920), phospho‐ERK (Cell Signalling – #9106), ERK (Santa Cruz – ERK2 (C‐14):sc‐154), ETV1 (Sigma‐HPA077249), ERG (Abcam – ab92513), GAPDH (Santa Cruz – (6C5):sc‐32 233) and alpha tubulin (Santa Cruz – (B‐7): sc‐5286).

### 2.6 ELISA

Cell supernatants were collected for further measurements. HGF levels were assessed using a commercially available ELISA kit according to the manufacturer's instructions (Human HGF instant ELISA Kit, Invitrogen, Waltham, MA, USA, ref: BMS2029INST). Data are expressed as pg⋅mg^−1^ of total proteins.

### 2.7 RNA interference

For silencing, 400 000 cells were incubated with Lipofectamine 2000 (Invitrogen) mixed with 60 nM of Stealth RNAi™ siRNA Negative Control Lo GC (Invitrogen, REF: 462002) or a pool of Stealth RNAi™ siRNAs targeting MET receptor (Invitrogen, REF: 90695450). The cells were then plated in a 6‐well plate in a final volume of 1.5 mL of complete medium. The same batch of transfected cells were trypsinised after 48 h and used for experiments.

### 2.8 Performing phenotypic tests using the IncuCyte ^®^ device

The IncuCyte^®^ System is an imaging system dedicated to taking photographs of living cells. It enables real‐time quantification of cell behaviour in a stable, controlled environment using the incubator. The images obtained are taken in high definition 24 h a day, automatically and non‐invasively, providing a detailed insight into the biological processes at work. For the subsequent experiment, cells were initially cultured for 24 h and then subjected to a 24‐h serum starvation with 0% FBS.

#### 2.8.1 Measurement of cell proliferation capacity using IncuCyte ^®^

Cells are seeded in a 96‐well plate with standard culture medium during 24 h. Using the IncuCyte^®^, photographs were taken simultaneously in the 96 culture wells 24 h after seeding and starvation with 4000 cells per well and the entire well was monitored for 72 h to 96 h. Cell proliferation results were compiled from a minimum of 32 wells per condition. The graph of the compiled results was obtained from the cell density found at the bottom of the wells.

#### 2.8.2 Measurement of cell migration capacity using IncuCyte ^®^

Using an IncuCyte^®^ claw device, wounds in the cell mat were made simultaneously in the 96 culture wells 48 h after seeding and starvation with 35 000 cells. The wound area was monitored for 48 h. Results were compiled from a minimum of 32 wells per condition with one claw in each well. The graph of compiled results was obtained from the cell density found at the bottom of the wells.

### 2.9 Measurement of cell migration and invasion capacity in Boyden chamber

Invasion inserts (“Growth Factor Reduced – Corning^®^ Matrigel^®^ Invasion Chamber”; Corning^®^, Amsterdam, Netherlands; REF: 354483) coated with Matrigel^®^ were equilibrated with 10% FBS medium 2 h before use. Cells were seeded in the upper chamber of the equilibrated invasion inserts or migration inserts (“Cell Culture Insert – Transparent PET Membrane, 8.0 μm pore size”; Corning^®^; REF: 353097) at 150 000 cells per well for PC3M and 135 000 cells for PC3 in 0% FBS medium. The lower chambers were loaded with 500 μL of 10% FBS medium to create a chemoattractant environment. When indicated, cells were first treated for 1 h with Capmatinib and then stimulated with HGF. Cells were incubated for 24 h at 37 °C in a CO_2_ incubator. After removing the cells from the upper surface with a cotton swab, the cells were fixed with cold methanol and stained with Hoechst at 5 μg⋅mL^−1^ (Sigma Aldrich Chimie, Saint Quentin, France, REF: H33258). Cells that had invaded or migrated through the gel were found on the lower surface and counted using an imagej program based on microscopy images.

### 2.10 Cell viability analysis

3‐(4,5‐Di‐2‐yl)‐2,5‐ditetrazolium bromide (MTT) assay was performed to evaluate the cytotoxic effect of Capmatinib (Euromedex SE‐S2788). The cells were seeded in 96‐well plates (15 000 cells⋅well^−1^), cultured for 24 h and treated with Capmatinib (0.25–10 μm). Then, the cells were incubated with 1 mg⋅mL^−1^ MTT solution during 2 h at 37 °C then solubilised with HCL/Isopropanol during 30 min. The absorbance was measured at 450 nm with Multiskan RC Thermo Labsystem.

### 2.11 Immunofluorescence

Cell samples were collected and fixed in 5% paraformaldehyde (PFA) for 15 min at room temperature. Samples were then washed three times with phosphate‐buffered saline (PBS) for 5 min each. Permeabilisation was achieved by incubating samples in 0.1% Triton X‐100 for 10 min. Samples were blocked with 1% bovine serum albumin (BSA) in PBS for 1 h to reduce non‐specific binding. Primary antibodies specific to the target protein were diluted in blocking solution and incubated with samples overnight at 4 °C. MET (Santa Cruz, REF: (C‐12): sc‐10), ETV1 (Sigma, REF: HPA077249) and ERG (Abcam, REF: ab92513). After primary antibody incubation, samples were washed three times with PBS. Appropriate secondary antibodies conjugated to fluorescent dyes were applied to samples (Goat anti‐Mouse IgG (H + L) Highly Cross‐Adsorbed Secondary Antibody, Alexa Fluor™ 488, Thermo Scientific™, REF: A11029; Goat anti‐Rabbit IgG (H + L) Highly Cross‐Adsorbed Secondary Antibody, Alexa Fluor™ 488, Thermo Scientific™, A11034; Donkey anti‐Goat IgG (H + L) Cross‐Adsorbed Secondary Antibody, Alexa Fluor™ 488, Thermo Scientific™, A11055) and incubated for 1 h at room temperature. Nuclei were stained with DAPI (4′,6‐diamidino‐2‐phenylindole) (Sigma, REF: MDB0015‐10 mL) for 5 min. Samples were mounted with an anti‐fade mounting medium on glass slides. Fluorescent signals were visualised using a fluorescence microscope or confocal microscope at the appropriate excitation and emission wavelengths. Quantitative analysis of fluorescence intensity or co‐localisation was performed using zen software.

### 2.12 Histology and immunochemistry

Animal tissues were fixed in 4% paraformaldehyde, paraffin‐embedded, and histological sections of 5 μm thickness were mounted on tissues slides. Briefly, slides were deparaffinised and rehydrated by standard protocol. Antigen retrieval was performed by heating until boiling for 4 min 30 s in sodium citrate 10 mm pH 6.0 or Tris EDTA Tween 20 0.5% and the cooled down at room temperature for 40 min, followed by 1 h of protein blocking using a solution of BSA 5%, donkey sera 5%, Triton X100 0.3% to prevent non‐specific binding. Then, slides were incubated overnight at 4 °C, with primary antibodies. The goat anti‐Human HGF R/cMET (AF276, R&D Systems, USA) the rabbit anti‐ Phospho‐Met (Tyr1234/1235) (#3126; Cell Signaling Technology, MA, USA), the mouse anti‐Vimentin (M0725; Dako, Santa Clara, CA, USA) were used at a dilution 1:50. The rabbit anti‐PCNA ab 18 197 from Abcam, US, was used at a dilution 1:1000. The rabbit anti‐Cleaved Caspase 3 active #9661S, Cell Signaling Technology, was used at a dilution 1:500. After several washings, the primary antibodies were coupled with Alexa Fluor 488 antibody for 1 h at the dilution 1:500 (Alexa Fluor 488 Goat anti‐mouse A11029, Alexa Fluor 488 Goat anti‐rabbit A11034, Alexa Fluor 488 Donkey anti‐Goat A11055, Thermo Fischer Scientific, Waltham, MA, USA). Then, nuclei were dyed with DAPI 1/5000 for 10 min (SIGMA; ref MBD0015) and slides were mounted in mounting medium (S302 3802; Agilent, Santa Clara, CA, USA) covered with a glass coverslip with nail and stored in the dark at 4 °C until imaged. Images were acquired with a Zeiss Axioscan Z1 slide scanner (Zeiss, Rueil Malmaison, France) or Zeiss Imager‐Z1 at 20× to 40× magnification. All images were minimally processed using Zeiss Zen^®^ (Zeiss) and fiji software. Image analysis was performed by fiji. IHC quantification was carried out using a macro established by the BiCel platform based on the following principle: quantification of the number of nuclei in a field using imagej and measurement of the average intensity in the field using zen software. The operation is repeated until all the fields cover the slice.

The average intensity per nucleus is then calculated and the results expressed as MET intensity per nucleus. Quantification was performed on 4 to 10 slides containing 5–9 sections.

### 2.13 Animal studies and approval

The project and experimental protocols received an ethical approval by the French Committee on Animal Experimentation and the Ministry of Education and Research (approval number 19253‐201903191709966 v1). All experiments were performed in accordance with relevant guidelines and regulations. Mice were bred under SOPF conditions at the Animal Research Laboratory of Institut Pasteur de Lille. Experiments were performed in an isolator with six mice housed in M‐BTM cage (Innovive) and allowed to eat and drink *ad libitum*. PC3M cells (1 × 10^6^) in PBS were injected subcutaneously into both flank of 8‐week‐old male NSG‐hHGFki mice, obtained from Charles River Laboratories (NOD.Cg‐Hgftm1.1(HGF)Aveo Prkdcscid Il2rgtm1Wjl/J – RRID: IMSR_JAX:014553). NSG‐hHGFki mice are NOD.scid. Il2Rγcnull (“NSG”) animals with the Hgftm1.1(HGF)Aveo “humanised” knock‐in allele (hHGFki). Homozygous NSG‐hHGFki mice have no mature T cells or B cells, lack functional natural killer (NK) cells, are deficient in cytokine signalling, and express human hepatocyte growth factor (HGF) in place of the endogenous mouse HGF. The half of the mice were treated by oral gavage 5 days a week by Capmatinib (10 mg⋅kg^−1^⋅day^−1^; MedchemExpress HY‐13444) directly after subcutaneous injections. Capmatinib was suspended in DMSO for cell treatment, and a vehicle containing 0.5% methylcellulose and 0.1% Tween80 in water, sonicated and brought to a pH of 2.5–3.5 for *in vivo* use. The evolution of the tumoral growth was followed by palpation and weighing. Intra‐ and inter‐cage behaviour was also checked. This mouse model needs to be bred and handled in an isolator because of its fragility. The studies were stopped before the reaching of threshold distress or suffering of the mice. After the mice were euthanised, the tumours were removed for analysis.

### 2.14 Next‐generation sequencing

#### 2.14.1 Construction of sequencing libraries

The 3′RNA‐seq transcriptome was performed following the steps described in [47] but starting from 200 ng of total RNA and performing 14 cycles of amplification.

#### 2.14.2 Sequencing

Each library is pooled equimolarly and the final pool, after being controlled on Agilent bioanalyzer 2100, is sequenced on a NovaSeq 6000 (Illumina) with 100 cycles chemistry.

#### 2.14.3 Data analysis for transcriptomic data

The data processing steps are the same as those described in [47], except that the alignment was performed on GRCh38. Differential Gene Expression of RNA‐seq was performed with R/Bioconductor package DESeq2. The cut‐off for differentially expressed gene was *P*‐value *P*_adj_ (BH) < 0.05.

### 2.15 PANTHER 18.0 classification system

GO analysis for biological processes was carried out using the Gene Ontology resource (http://geneontology.org). For investigating genes ontology and properties, PANTHER version 18.0 (https://pantherdb.org) was used. Transcriptomic profiles corresponded to representation of gene categorisation into various groups, characterised in the literature and public data. Outputs from classification of shared “biological process” and “biological regulation” were exported as .txt files into Microsoft Excel and used to generate the associated figures.

### 2.16 Statistical analysis

All data are presented as mean ± SD, unless otherwise indicated in the figure legend. The analysis of differences between groups was performed with GraphPad Prism 8.0 for one‐way or two‐way ANOVA. *P* < 0.05 was considered a statistically significant difference (*P < 0.05; **P < 0.005; ***P < 0.0005; ****P < 0.00005).

## 3 Results

### 3.1 MET signalling relays ETS factors expression

**Fig. 1:**
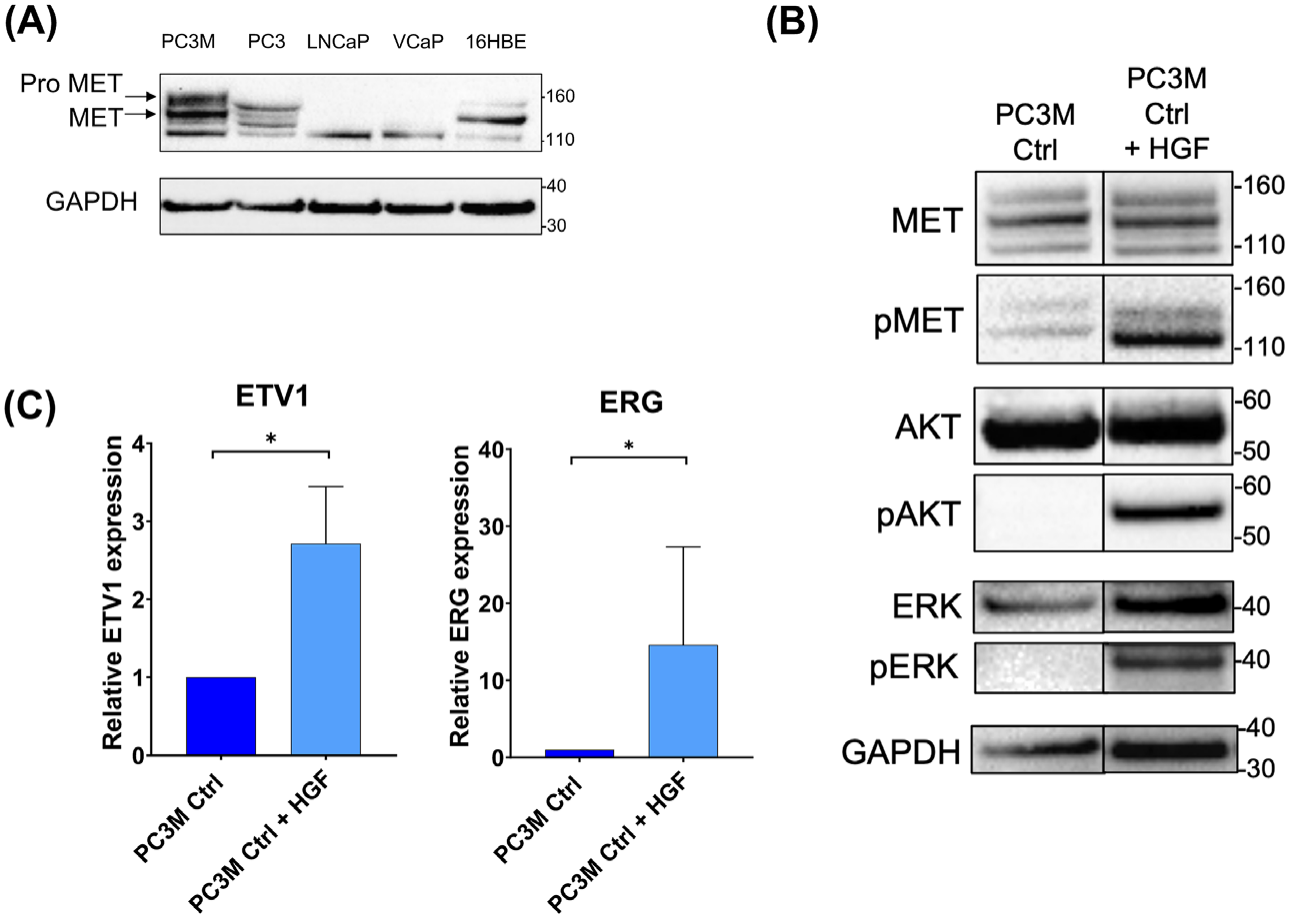
MET expression and signalling pathway activation. (A) For each cell line PC3M, PC3, LNCaP, VCaP and 16HBE (as control for MET expression), the same amount of protein was analysed by western blotting using an antibody directed against MET (upper panel). Pro MET and MET are indicated by an arrow. Expression was compared with the reference protein GAPDH (lower panel) (*n* = 3). (B) Protein expression of MET, pMET, AKT, pAKT, ERK and pERK was analysed by western blot in PC3M Ctrl cell lines stimulated or not by HGF during 30 min. The membrane was first probed with antibodies directed against pMET, pAKT, pERK and reprobed sequentially using an anti‐ MET, AKT and ERK antibodies on each corresponding membrane. Expression was compared with the reference protein GAPDH (*n* = 3). (C) The transcriptional expression of ETV1 and ERG was analysed by RT‐qPCR in PC3M Ctrl stimulated or not by HGF during 24 h. Expression was normalised to the expression of the reference gene *TBP* (*n* = 3). Data are means of 4 experiments for ETV1 expression (*n* = 4) and 3 for ERG expression (*n* = 3) analysed by two‐way ANOVA ± SEM. *P < 0.05.

To investigate the interplay between ETV1, ERG and MET signalling, we selected prostate cancer cell lines expressing MET endogenously, the PC3M and PC3. These PCa cells have a hormone‐independent status and present aggressive properties, even more pronounced for PC3M. The MET expression was evaluated and confirmed through western blot by the presence of mature and cleaved forms of the receptor in these cells in comparison to two hormone‐dependant cell lines, VCaP and LNCaP, not expressing MET and a control line, 16HBE, already characterised for its MET expression (Fig. 1A).

We used HGF stimulation to assess ETV1, ERG and MET expression and MET activation. Firstly, we demonstrated that MET signalling could be activated in PC3M and PC3 cells, as shown by western blot in Fig. 1(B) and Fig. S1(A), informed by the autophosphorylation of MET and the phosphorylation of its downstream AKT and ERK kinases under HGF stimulation. We then checked the expression of ETV1 and ERG in the different conditions. As depicted in Fig. 1(C), HGF stimulation induced a significant increase in ERG and a smaller increase in ETV1 in PC3M. This up‐regulation was also observed in PC3 cells (Fig. S1B).

These results suggest that MET signalling mediates ETV1 and ERG expression, raising the question of a reciprocal stimulation in our models of prostate cancer.

### 3.2 ETS factors overexpression regulates MET expression and activity

**Fig. 2:**
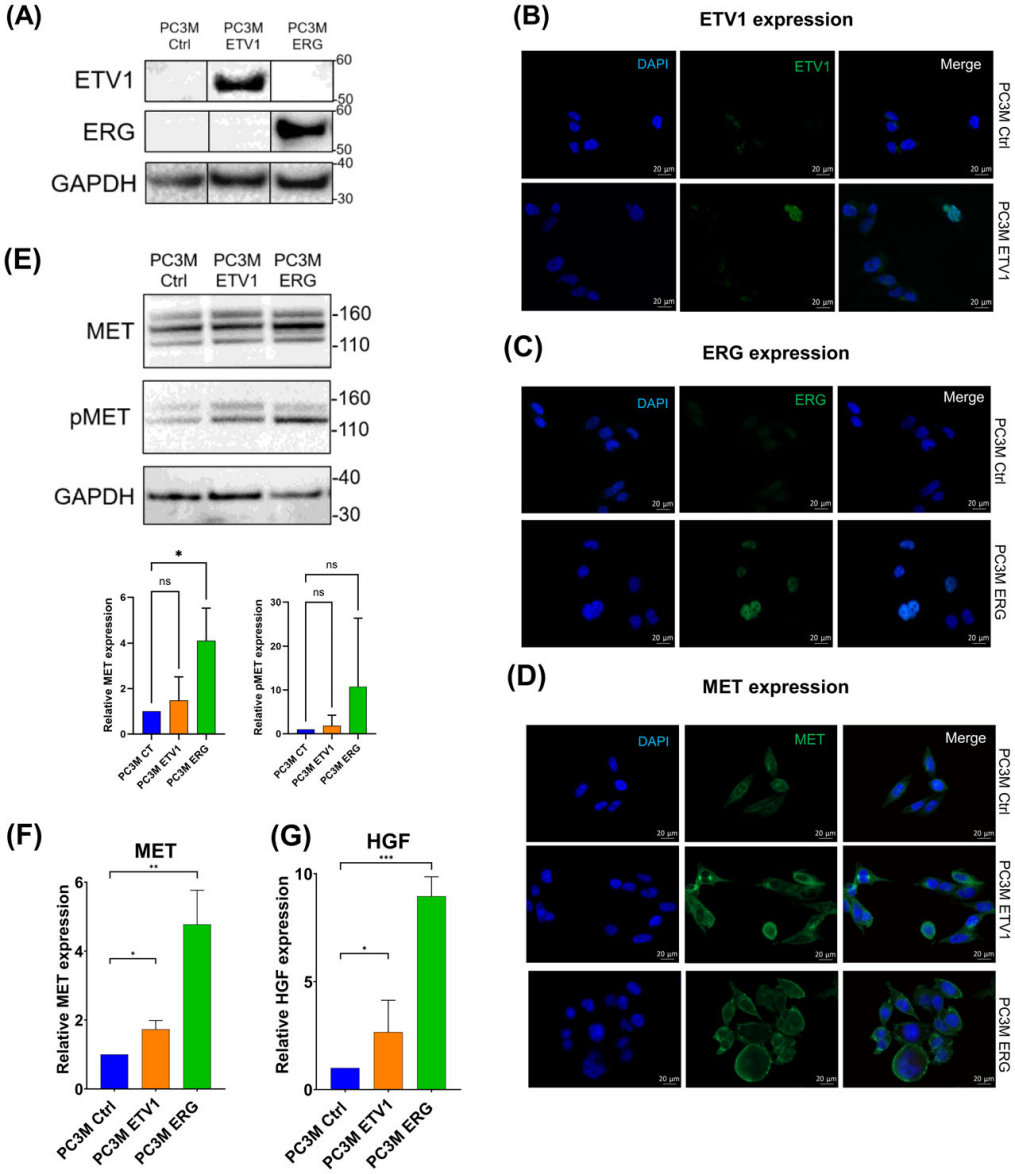
Analysis of ETV1, ERG, MET and HGF expression in established PC3M cells overexpressing ETV1 and ERG. (A) Protein expression of ETV1 (upper panel) and ERG (middle panel) was analysed by western blot in PC3M Ctrl, PC3M ETV1 and PC3M ERG cell lines. Expression was compared with the reference protein GAPDH (lower panel). Data are means of 3 experiments (*n* = 3) and analysed by one‐way ANOVA ± SEM. (B–D) Protein expression of ETV1 (B), ERG (C) and MET (D) was detected by immunofluorescence in PC3M Ctrl, PC3M ETV1 and PC3M ERG cell lines (*n* = 3). Expression was compared with nuclear staining using DAPI. The merge signal was illustrated in the panels of the right. Scale bar 20 μm. (E) Protein expression of MET (upper panel) and pMET (middle panel) was analysed by western blot in PC3M Ctrl, ETV1 and ERG cell lines. The membrane was first probed with anti‐ pMET antibody and reprobed sequentially using an anti‐MET antibody. Expression was quantified and compared with the reference protein GAPDH (lower panel). Data are means of 5 experiments (*n* = 5) and analysed by one‐way ANOVA ± SEM. (F, G) Transcriptional expression of *MET* (F) and *HGF* (G) was analysed by RT‐qPCR in PC3M Ctrl, PC3M ETV1 and PC3M ERG cell lines. Expression was normalised to the expression of the *TBP* reference gene (*n* = 3). Data are means of 3 experiments (*n* = 3) analysed by two‐way ANOVA ± SEM. ns, non‐significant, *P < 0.05; **P < 0,005; ***P < 0.0005.

To explore the regulation link between MET and ETV1/ERG, we performed an overexpression of ETV1 or ERG by retroviral infection. After establishment of the cell lines, overexpression of ETV1 and ERG was validated by western blot and immunofluorescence as shown in Fig. 2(A–C) for PC3M and Fig. S2(A–C) for PC3, in comparison with the corresponding control cells.

We then studied the expression level and activity of MET and its high‐affinity ligand, HGF. We observed that MET expression was increased after ETV1 and ERG overexpression, as shown by western blot and a relative quantification (Fig. 2E for PC3M and Fig. S2E for PC3), immunofluorescence (revealing predominately membrane localisation for MET – Fig. 2D for PC3M and Fig. S2D for PC3) and RT‐qPCR (Fig. 2F for PC3M and Fig. S2F for PC3).

We also examined HGF expression by RT‐qPCR and showed that overexpression of ETV1 and ERG resulted in a three‐ to eight‐fold increase in HGF expression in PC3M (Fig 2G) but not statistically significant in PC3 (Fig. S2G). Nevertheless, the secretion of HGF, tested by ELISA in PC3M and PC3 cells (Fig. S3), was not detectable after ETV1 or ERG overexpression.

Taken together, these results, obtained in two hormone‐independent cell lines, show that ETV1/ERG and MET signalling are intrinsically linked.

### 3.3 ETS factors enhance pro‐migratory and pro‐invasive effects intensified by MET activation

**Fig. 3:**
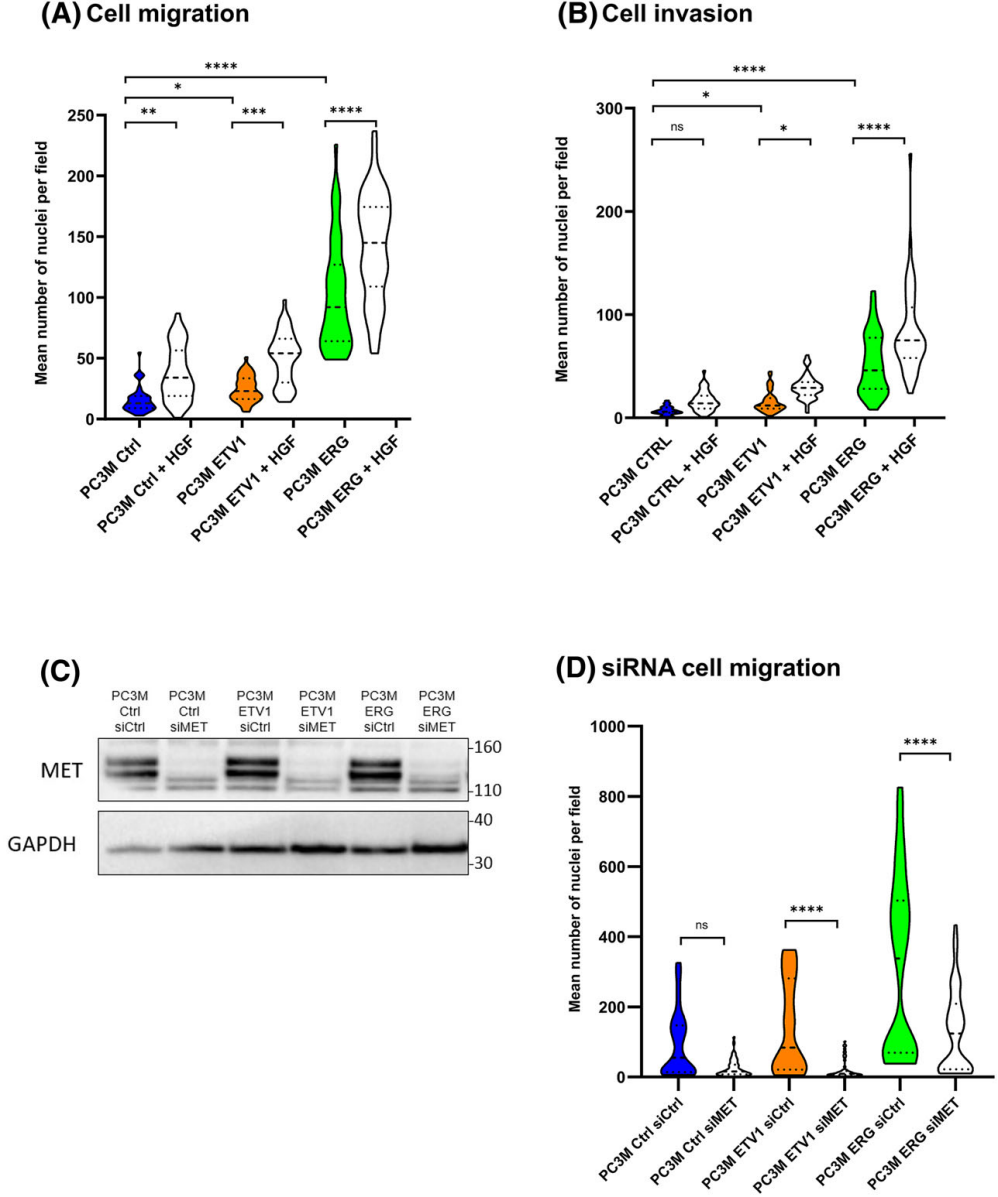
Migration and invasion capacities of ETV1 and ERG overexpressing PC3M cells treated or not by HGF or after MET silencing. (A) Migration tests were carried out in a Boyden chamber by measuring the number of nuclei passing through the membrane after 24 h for PC3M cells treated by HGF or not (*n* = 3) represented by the panel under the graph. Data are means of 3 experiments (*n* = 3) analysed by one‐way ANOVA ± SD represented by violin plot. (B) Invasion tests were carried out in a Boyden chamber by measuring the number of nuclei passing through the membrane after 24 h for PC3M cells treated by HGF or not represented by the panel under the graph. Data are means of 3 experiments (*n* = 3) analysed by one‐way ANOVA ± SD represented by violin plot. (C) Protein expression of MET was analysed by western blot in PC3M Ctrl, ETV1 and ERG cell lines after siRNA treatments (siCtrl and siMET) during 24 h. For each conditions, the same amount of protein was analysed by western blotting using an antibody directed against MET (upper panel). Expression was compared with the reference protein GAPDH (lower panel) (*n* = 3). (D) Migration tests were carried out in a Boyden chamber by measuring the number of nuclei passing through the membrane after 24 h for PC3M cells transfected by siCtrl or siMET during 48 h before (*n* = 3) represented by the panel under the graph. Data are means of 4 experiments (*n* = 4) analysed by one‐way ANOVA ± SD represented by violin plot. ns: non‐significant, *P < 0.05; **P < 0.005; ***P < 0,0005; ****P < 0.00005.

To investigate the involvement of ETV1, ERG and MET in pro‐tumour capacities and the effect of their concomitant activity, we assessed migration and invasion using Boyden chamber assays, comparing all cell lines. We showed in Fig. 3(A,B) for PC3M and Fig. S4A,B for PC3, that ETV1 (violin bar in orange) and ERG (in green), significantly promote migration and invasion in comparison to Ctrl cells (in blue). When MET is activated by HGF, the same modulations were observed on control cells and the migratory and invasive properties induced by ETV1/ERG were further amplified. To determine whether these effects could be reversed by MET inhibition, we performed a migration assay using small interfering RNA (siRNA) against MET, after checking the effectiveness of inactivation by siMET. As shown in the western blot in Fig. 3(C), MET repression is effective with an almost complete reduction of MET in all cell lines. MET repression significantly reduced migration in cells overexpressing ETV1 or ERG, whereas repression was more limited in PC3M control cells (Fig. 3D). Similar results were found for PC3 cells (Fig. S4C,D).

We also carried out experiments measuring proliferation in order to study the impact of ETV1, ERG and MET stimulation on this cellular property. As shown in Fig. S5, neither ETV1 nor ERG induced perceptible alterations in PC3M and PC3 cell proliferation, and HGF stimulation of MET did not result in significant changes.

Overall, these results suggest that ETV1 and ERG, as well as MET activation, play an important role in prostate cancer cell migration and invasion, without affecting proliferation, and that MET receptor activation leads to an increase in these responses and MET repression to reversion.

### 3.4 ETS factors induce pro‐tumorigenic activity in mice, reversed by MET inhibition with Capmatinib treatment

To elucidate the significance of the ETV1/ERG/MET collaboration in tumorigenesis, we conducted experiments based on an NSG‐hHGFki mouse model, in which the human HGF knock‐in gene replaces the murine HGF gene. As murine HGF cannot activate the human MET receptor, we used this humanised mouse model expressing human HGF to assess tumour progression using PC3M cells. The presence of human HGF in these mice enables the activation of human MET on the surface of injected human cells. We performed subcutaneous injection of PC3M Ctrl, ETV1 and ERG cells into both flanks of the mice and monitored tumour growth over one month. To report on the interface between ETV1/ERG and MET signalling in this mouse model, mice were also subjected to oral gavage 5 days a week with Capmatinib, a tyrosine kinase inhibitor (TKI) that specifically binds to the MET receptor. Efficacity of Capmatinib inhibition was evaluated in PC3M cells prior to these trials. As shown on the western blot analysis of Fig. S6, the autophosphorylation of MET and phosphorylation of downstream AKT and ERK kinases are blocked by Capmatinib treatment with doses as low as 10 nm (Fig. S5A for the dose range on PC3M ctrl cells and Fig. S5B for all cell lines at 10 nm). Capmatinib toxicity was also tested through MTT tests at higher concentration (Fig. S6C). We assessed the impact of Capmatinib on migration and showed that Capmatinib significantly reduced the migratory capacity of cells (Fig. S7).

**Fig. 4:**
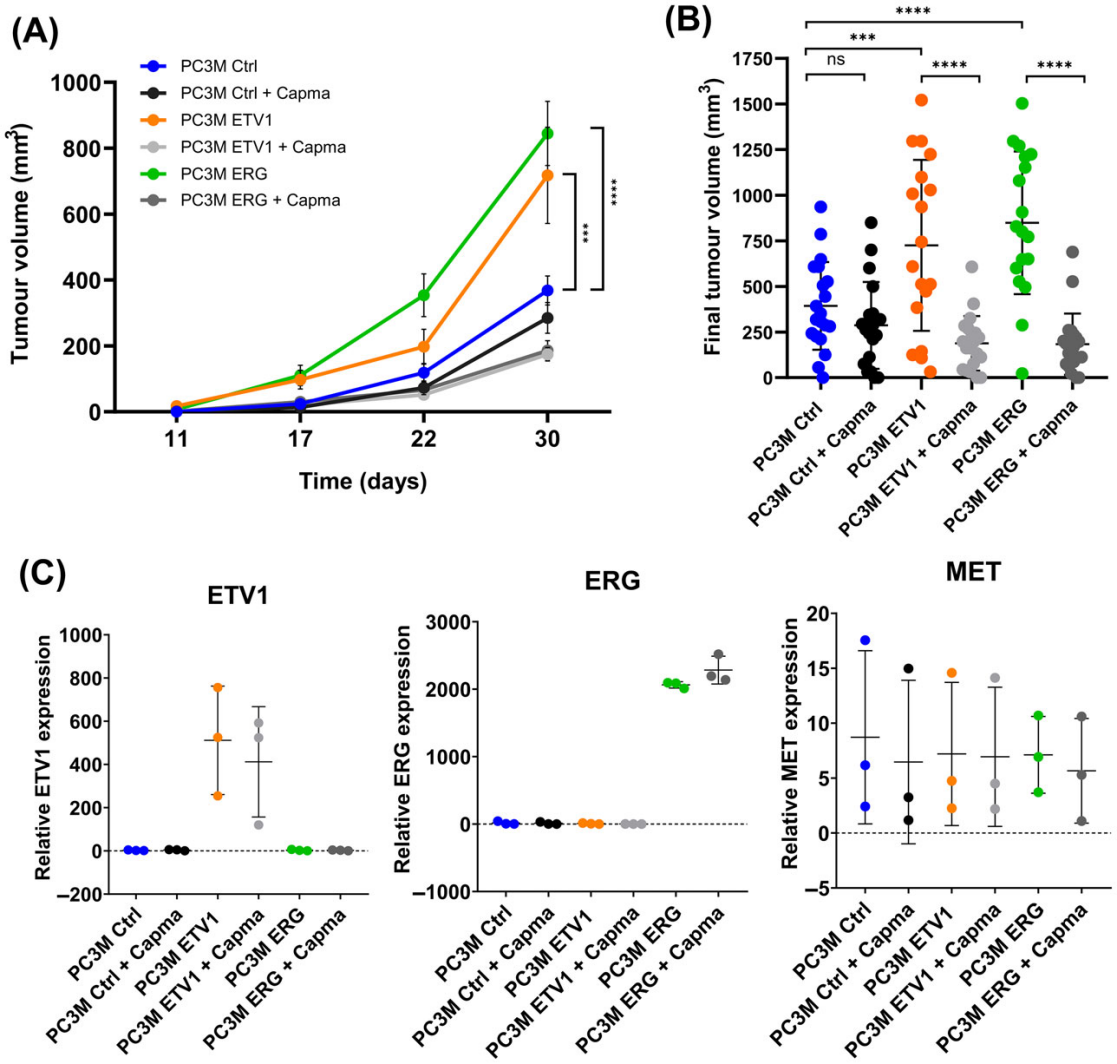
Tumour growth capacity of ETV1 and ERG overexpressing PC3M cells in NSG‐hHGFki mice and after Capmatinib treatment. (A) Graphical representation of tumour volume over time after subcutaneous injection of PC3M Ctrl, PC3M ETV1 and PC3M ERG cells. The tumour volume of the mice was monitored by palpation. Data are means of the tumours volume of 10 mice by condition analysed by one‐way ANOVA ± SD. (B) Graphical representation of the final tumour volume of mice having been treated or not with Capmatinib. Data are means of the tumours volume of 10 mice by condition analysed by one‐way ANOVA ± SD. (C) The transcriptional expression of *ETV1*, *ERG* and *MET* was analysed by RT‐qPCR in mouse tumours induced by the PC3M Ctrl, PC3M ETV1 and PC3M ERG cell lines. Expression was normalised by the expression of the *TBP* referent gene (*n* = 3). Data are means of 3 experiments (*n* = 3) analysed by two‐way ANOVA ± SD. ns, non‐significant, ***P < 0.0005; ****P < 0.00005.

Tumour growth evolution depicted in Fig. 4(A) indicated that overexpression of ETV1 and ERG resulted in tumour volumes two to three times greater than those observed for the PC3M Ctrl cells. Administration of Capmatinib resulted in a significant reduction in tumour volumes, which reached levels close to those of control tumours. The comparison of final tumour volumes between treated and untreated conditions revealed that Capmatinib induced a reduction in tumour volume only in mice injected with the PC3M ETV1 and PC3M ERG cell lines and not in control mice (Fig 4B).

From the tumours, we extracted RNA and verified the expression of ETV1, ERG and MET using RT‐qPCR. As shown in Fig. 4(C), Capmatinib treatment did not induce any alteration in the expression of ETV1, ERG or MET.

**Fig. 5:**
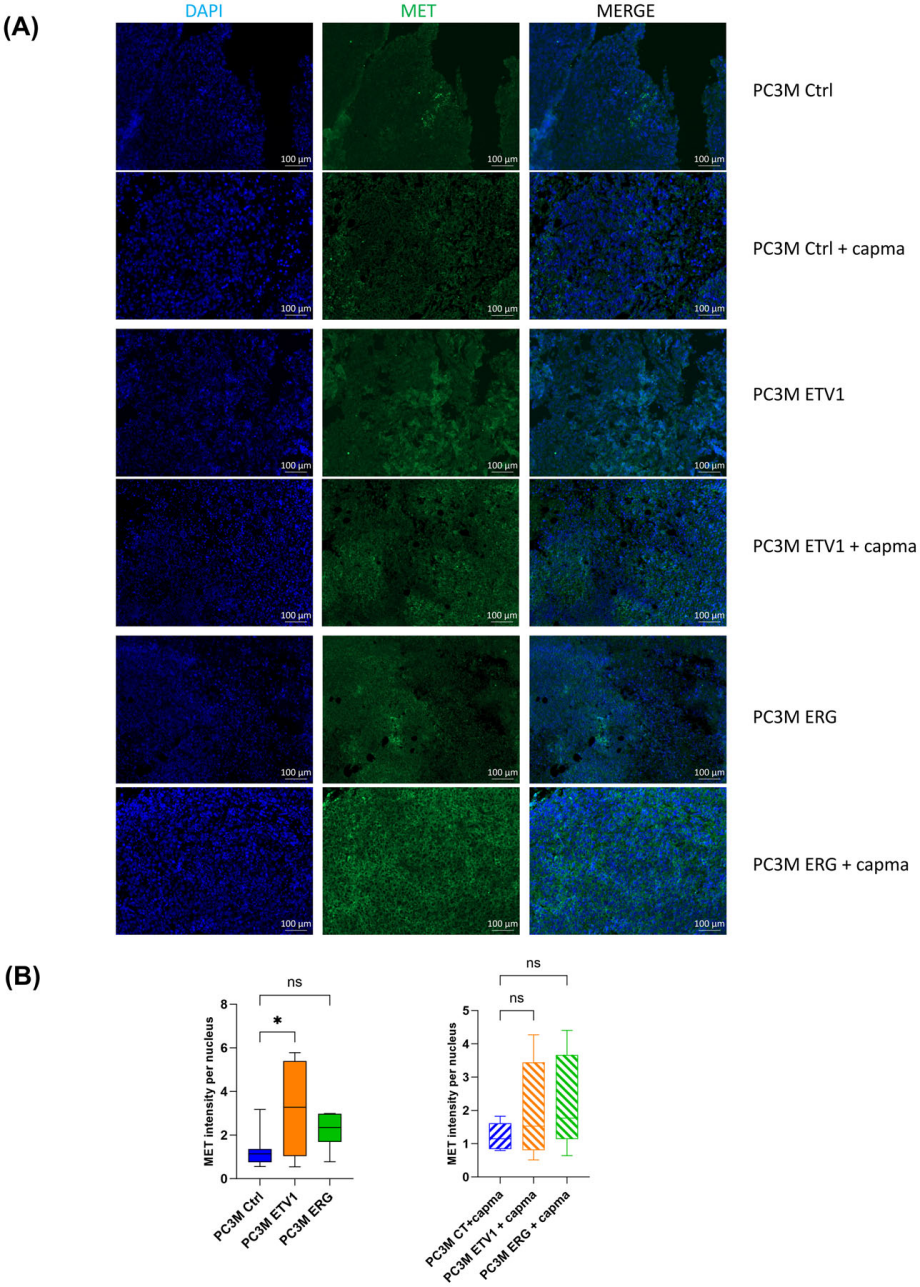
MET expression by immunochemistry in control, ETV1 and ERG tumours. (A) MET immunostaining in PC3M Ctrl, PC3M ETV1 and PC3M ERG tumours treated or not with Capmatinib. Expression was compared with nuclear staining using DAPI. The merge signal was illustrated in the panels of the right (*n* = 3). Scale bar 100 μm. (B) Quantification of MET immunostaining in PC3M Ctrl, PC3M ETV1 and PC3M ERG tumours treated or not with Capmatinib. Quantification was carried out by one‐way ANOVA ± SD (*n* = 3). ns, non‐significant, *P < 0.05.

We also performed immunohistochemistry on tumour sections for MET and phosphoMET which showed expression in control, ETV1 and ERG tumours with a statistically representative increase in ETV1 tumours and slight increase in ERG tumours, as shown in Fig. 5 for MET and Fig. S8 for phosphoMET. Capmatinib treatment revealed a decrease in phosphoMET detection, that is in line with the reversion of MET activity, even if the data are not statistically confirmed.

PCNA analysis and quantification showed a downward trend in TKI‐treated control‐ETV1 or ‐ERG mice, which is consistent with a potential reduction in proliferation in the treated tumour and therefore with a reduction in tumour volume (Fig. S9).

Our *in vivo* results are therefore consistent with the *in vitro* experiments and suggest that not only do ETV1 and ERG promote the formation of larger tumours, but that MET also participates in these effects, notably observed by the reversal of ETV1/ERG‐induced tumour progression by Capmatinib.

### 3.5 Transcriptomic analysis supports similarities between ETS factors and reveal MET collaboration target genes signature

In order to identify a distinctive molecular signature of ETV1/MET or ERG/MET cooperation, transcriptomic analysis was performed using high‐throughput 3′ RNA sequencing on samples of PC3M Ctrl, ETV1 and ERG cell lines stimulated or not by HGF. Genes were sorted based on their respective *P*‐values, and those exhibiting values equal to or <0.05 were retained for further analysis.

Based on this threshold and a cut‐off of 1.5 for fold change, we obtained five lists of target genes by comparing the Ctrl *versus* ETV1 or ERG or HGF lists and the Ctrl‐HGF *versus* ETV1– or ERG + HGF lists (named ETV1, ERG, HGF, ETV1 + HGF and ERG+HGF lists), deposited in the NCBI SRA database. These various comparisons have made it possible to define genes that are specifically regulated, but above all genes that are common to ETV1, ERG and/or stimulated HGF‐MET, and in particular those involved in their association. We also performed more global analyses by using PANTHER 18.0 classification system, thereby facilitating gene ontology and subsequent classification based on their properties, yielding gene expression profiles. Classification thus makes it possible to define categories and sub‐categories, and these sets can be compared for the different experimental conditions.

#### 3.5.1 ETV1 and ERG factors present similar transcriptomic profile as HGF stimulation

**Fig. 6:**
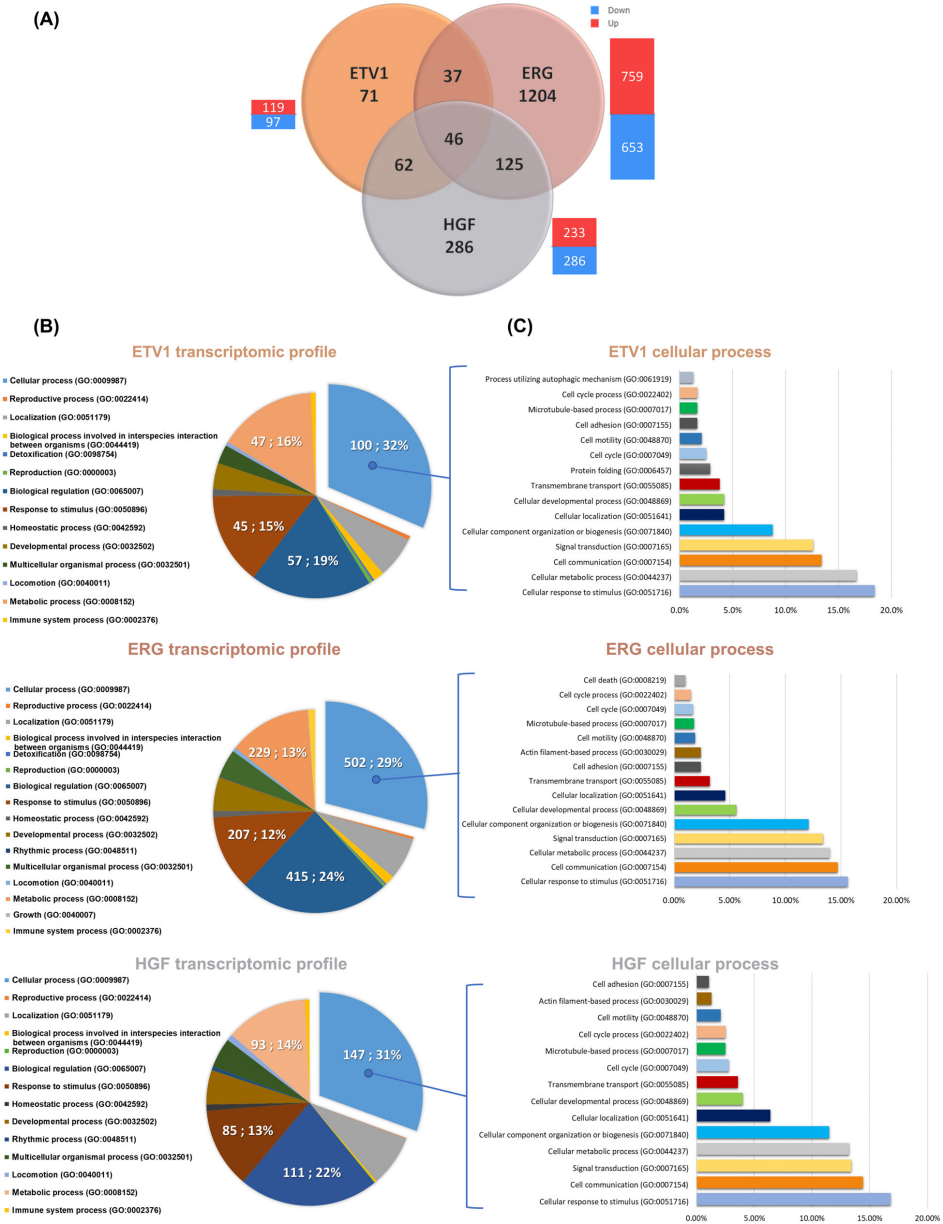
Gene ontology (GO) enrichment of differentially regulated genes in ERG, ETV1 and HGF‐stimulated PC3M models. (A) Genes were selected by *P* value ≥0.05 and fold change ≤1.5 and compared in a Venn diagram representation of differential and common genes expression between PC3M ETV1, PC3M ERG and PC3M stimulated by HGF compared to PC3M Ctrl. (B) Pie charts graphical representation of PC3M ETV1, PC3M ERG and HGF stimulated PC3M transcriptomic profiles elaborated with PANTHER 18.0 version. (C) “Cellular process” charts. Graphical representation of “cellular process” from the PANTHER classification sub‐categories in PC3M ETV1, PC3M ERG and HGF‐stimulated PC3M conditions.

Under these conditions, we first focused on PC3M ETV1 and ERG analyses. As shown in the Venn Diagram of Fig. 6(A), a total of 216 genes exhibited differential expression in the ETV1 group and 1412 genes in the ERG group, when compared to the PC3M Ctrl group. These genes showed a balanced distribution of both up‐regulated and down‐regulated expressions. 83 genes were found to be commonly regulated in both ETV1 and ERG groups (Fig. 6A). Interestingly, we found genes that were already well characterised as target genes for ETV1 or ERG, such as a number of *MMPs*, *BMPs* or *BCL2*, as illustrated in Table S2.

To investigate the distribution of regulated genes in the different classes of the biological processes, we performed a PANTHER 18.0 analysis of the global target gene lists for ETV1 and ERG. These data were used to obtain the “ETV1 transcriptome profile” and the “ERG transcriptome profile”, illustrated by the pie charts in Fig. 6(B). This resulted in 14 different categories for ETV1 and 16 categories for ERG, with the 14 common categories representing 99.8% of the genes. It is interesting to note that ETV1 and ERG therefore have identical transcriptome profiles repartition, with only a slight variation in proportions. Notably, these categories encompass four main categories representing more than 75% of the pie chart which are “cellular process” (±30%), “biological regulation” (±20%), “metabolic process” (±15%) and “response to stimulus” (±13%). The “cellular process” and “biological regulation” categories are the two most important, both in terms of percentage (50%) and in terms of relevance to the biological effects observed in our cellular models. Within the “cellular process” category, analysis by PANTHER 18.0 identified 15 sub‐categories for ETV1 and 15 sub‐categories for ERG, with a difference of 2 sub‐categories but representing <4% of the genes (Fig. 6C). The 13 common sub‐categories included classifications as “cell cycle”, “cell motility”, “cell adhesion”, “cellular developmental process”, “signal transduction”, “cell communication” and “cellular response to stimulus”. Here again, at this level of classification, ETV1 and ERG showed a very similar distribution of target genes. The same analysis was carried out for the “biological regulation” category and the same conclusions can be drawn. ETV1 and ERG shared the same 11 sub‐categories representing more than 95% of the regulated genes, which once again presented a very similar profile (Fig. S10).

To investigate the MET signalling impact on transcriptomic programme of PC3M cells and compare to those of ETV1 and ERG, we next explored the target gene list of PC3M cells stimulated by HGF. HGF stimulation induced 519 up‐ or down‐regulated genes, including 62 genes common to ETV1, 125 to ERG and 46 to both. Same as for ETV1 and ERG, we found genes that were already well characterised as target genes for MET (Table S2).

As previously described, we used PANTHER 18.0 to conduct a comprehensive assessment of the transcriptomic profile. As represented in Fig. 6(B), HGF stimulation revealed a transcriptomic profile repartition identical to that of ETV1 or ERG, with 14 previously described categories in common. Focusing on the “cellular process” (Fig 6C) and “biological regulation” (Fig. S10) categories, HGF stimulated‐condition shared in common with ETV1 and ERG, respectively 12 and 11 sub‐categories, corresponding to almost all the regulated genes.

Taken together, these results indicate that ETV1 and ERG factors and activation of MET signalling by HGF deploy a very similar transcriptomic programme with almost identical categories and sub‐categories of regulation.

#### 3.5.2 HGF‐induced MET signalling and ETV1/ERG share common highly regulated target genes involved in “cellular process” and “biological regulation”

In order to decipher more precisely the sets of genes found in the classifications, we looked for genes that are commonly and most strongly regulated by ETV1, ERG and HGF by setting a fold change threshold of 2.3 and for which the direction of regulation is identical.

229 genes were found in these conditions, with a number of genes common to both “cellular process” and “biological regulation” categories and their corresponding sub‐categories. These genes are depicted in the Excel file (Table S3) and classified according to the distribution obtained by PANTHER.

**Fig. 7:**
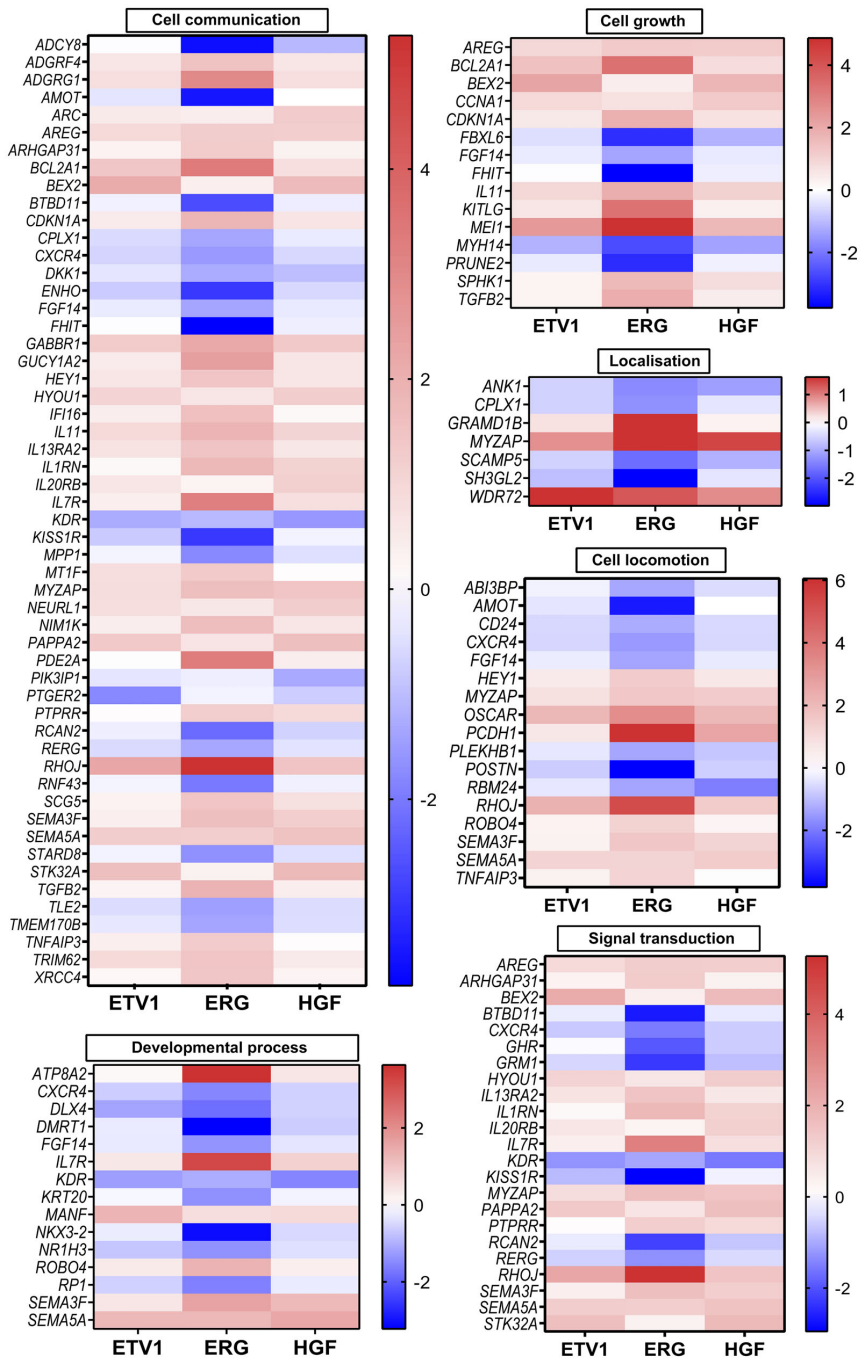
Heatmap of “cellular process” and “biological regulation” classification of common ETV1/ERG and HGF‐stimulated regulated genes. Heatmap representing the different combined subcategories of “cellular process” and “biological regulation” listed in cell locomotion, signal transduction, developmental process, cell growth, localisation and cell communication groups. Genes were selected by their fold change ≤2.3 and classified by alphabetic order according to the ETV1, ERG and HGF‐stimulated PC3M models.

Among them, 128 genes are up‐ or down‐regulated in the same direction for ETV1, ERG and also HGF. These common genes are shown in the heatmap of Fig. 7 in relation to their PANTHER classification in the corresponding categories and sub‐categories. Interestingly, numerous genes known to be implicated in migration, invasion or metastatic processes were retrieved, with some of them highly up‐ or down‐regulated, as for example *KITLG*, *TGFB2*, *IL11*, *AREG*, *SEMA3F*, *SEMA5A*, *OSCAR* or *FGF14*, *CXCR4*, *AMOT*, *PLEKHB1*, *CD24*, *GHR* and *RBM24*.

These results indicate that ETV1, ERG and HGF stimulation share regulated genes involved in processes related to migration, invasion, tumour growth and metastasis, some of which are quite highly regulated.

#### 3.5.3 Combination of MET activation and ETV1/ERG highlight relevant transcriptional signature

**Fig. 8:**
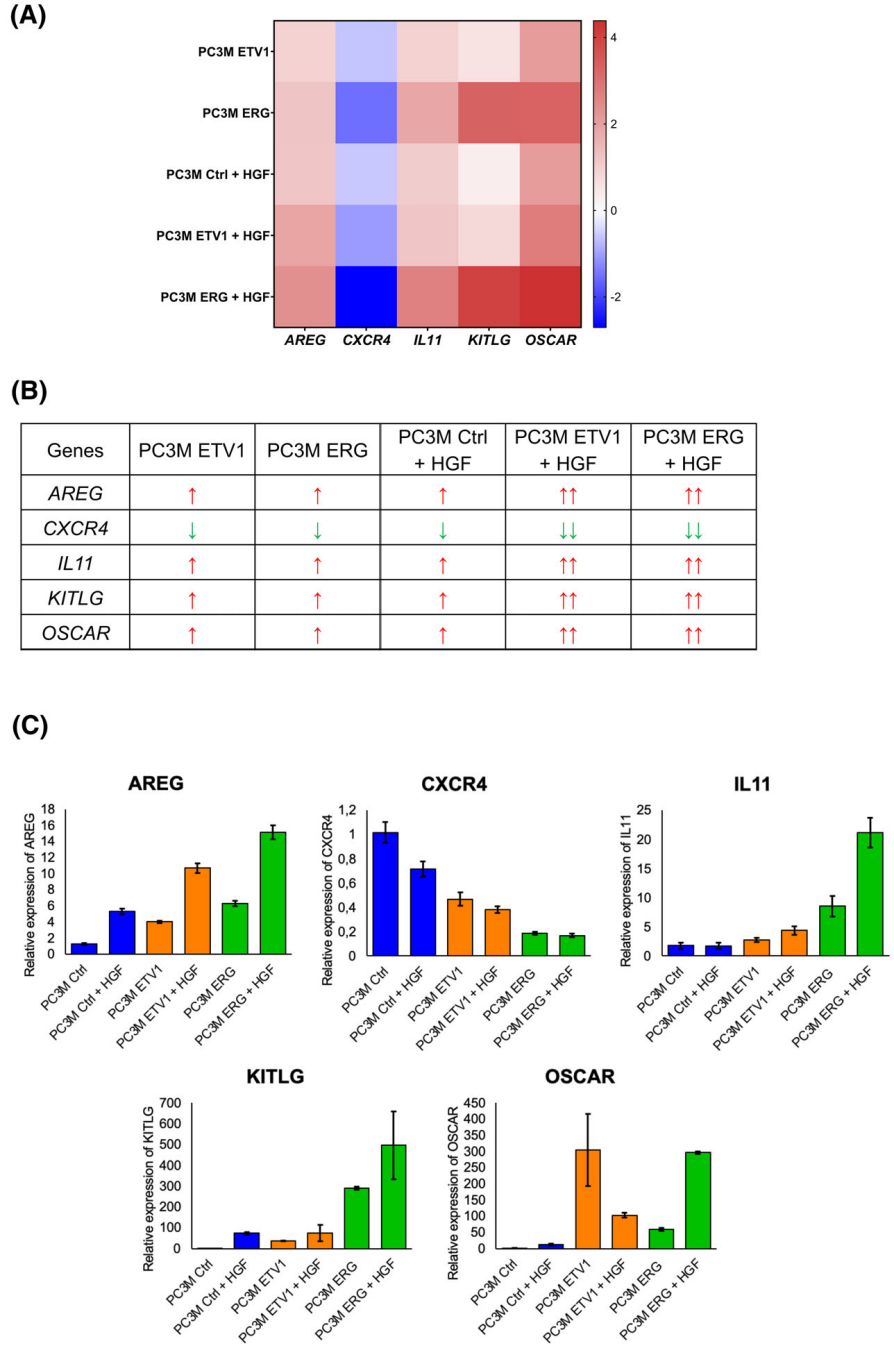
Transcriptomic analysis identification of specific regulated genes in the combined ETV1/ERG and HGF‐stimulated conditions. (A) Heatmap representation of the most highly regulated genes *AREG*, *CXCR4*, *IL11*, *KITLG* and *OSCAR* genes according to their log2foldchange value, in the different conditions (ETV1, ERG, HGF, ETV1 + HGF and ERG+HGF). Genes were classified by alphabetic order. Overexpressed genes are in red, down‐regulated genes in blue. (B) Visual representation of the direction and level of expression of *AREG*, *CXCR4*, *IL11*, *KITLG* and *OSCAR* regulated genes in all conditions (ETV1, ERG, HGF, ETV1 + HGF and ERG+HGF). (C) The transcriptional expression of *AREG* (*n* = 6), *CXCR4* (*n* = 4), *IL11* (*n* = 4), *KITLG* (*n* = 6) and *OSCAR* (*n* = 4) was analysed by RT‐qPCR in PC3M Ctrl, ETV1 and ERG stimulated or not by HGF during 24 h. Expression was normalised to the expression of the *TBP* reference gene. The error bars represent ± SD.

To explore the cooperation between ETV1/ERG and MET signalling at the transcriptional level, we looked for genes with significant regulation, found in the ETV1, ERG and HGF profiles but also in the ETV1 + HGF and ERG+HGF conditions, with the idea of highlighting a set of genes that represent a signature of the combinatorial action of the ETS and MET factors. For that, we took the most highly common regulated genes of the ETV1‐ERG‐HGF comparison and looked at their regulation in the ETV1 + HGF and ERG+HGF. Interestingly, on all the genes commonly found to be regulated under these conditions, five target genes stand out in this case, *AREG*, *CXCR4*, *IL11*, *KITLG* and *OSCAR*. The heatmap of Fig. 8(A) depicted the fold regulation of these genes in the five conditions and in Fig. 8(B) for a visual representation of the meaning and importance of regulating expression. We checked these modulations through RT‐qPCR and confirmed that genes are regulated in the same direction by ETV1, ERG and HGF alone and exhibited even more pronounced up‐ or down‐regulation in the ETV1 plus HGF or ERG plus HGF association (Fig. 8C). Thus, these five *AREG*, *CXCR4*, *IL11*, *KITLG* and *OSCAR* genes are relevant target genes that are synergistically regulated by ETV1/ERG and HGF and are good candidates for being a cooperation signature.

In summary, this comparative transcriptomic analysis sheds light on a relevant deciphering of the target genes regulated by the individual or combined action of ETV1/ERG and MET, offering potential prospects for the development of a specific molecular signature.

## 4 Discussion

Prostate cancer is currently treated by hormone therapies quickly leading to the resistance of the cancer and so on, to the formation of bone metastasis [48]. ETV1 and ERG fusions are well documented in prostate cancer and are present throughout disease stages, from initiation to metastasis spread [41, 49]. ETV1 fusions are detected in 10% of patients, while ERG fusions are present in 60%. These fusions arise from chromosomal rearrangements between androgen‐dependent promoters and ETV1 and ERG genes. Despite their regulation by androgen‐dependent promoters, they persist into hormone‐independent stages. MET is a potent oncogene down‐regulated by AR in prostate cancer, which explains its prevalence in advanced stages and bone metastases [50]. We therefore proposed that MET may be a relay in the expression of the ETV1 and ERG genes in hormone‐independent stages, leading to significant tumour capacity. This hypothesis is supported by literature, describing interesting collaboration between MET receptor and ETS factors. In fact, Gambarotta *et al*. showed that ETS1 is able to induce MET overexpression and in return MET induces ETS1 up‐regulation [51]. Over the years, a number of studies have described the participation of MET and ETS factors in various malignant tumours such as oesophageal cancer, lung cancer, melanoma, breast cancer and gastric cancer, and all refer to them as important in cancer progression and in particular as having a key role in cell proliferation, migration, invasion, apoptosis and the cell cycle [44, 46, 52, 53, 54]. In addition, studies have suggested that activation of MET receptor can induce the expression of various transcription factors from the ETS family such as ETS1 or the PEA3 group members (ETV1, ETV4, ETV5) through the MAPK signalling pathway [46, 51] and, in hepatocellular carcinoma, a regulation loop between ETV1 and HGF expression through MET pathways [43]. Thus, separately, ETV1, ERG and MET are well known to induce tumour properties in different cancers but their concomitant activity in prostate cancer is not yet described.

Using the PC3M and PC3 hormone‐independent prostate cancer cell lines, we explored the regulatory interaction between MET signalling and ETV1/ERG activity and demonstrated a reciprocal action link. Overexpression of ETV1/ERG led to an increase in MET receptor expression and activity (thanks to the fact that MET receptor is more basally phosphorylated in ETV1 and ERG presence) and to a slight elevated HGF mRNA expression which however do not lead to detectable secretion of HGF. Moreover, stimulation with HGF induced ETV1 and ERG expression. These results thus highlight MET crucial role in maintaining ETV1/ERG expression level and inversely, that of ETV1 and ERG in inducing MET expression.

After carrying out *in vitro* experiments, we demonstrated that ETV1 and ERG transcription factors induced migration and invasion capacities in PC3M and PC3 prostate cancer cells as well as HGF stimulation. The combination of the two showed that activation of the MET receptor amplified the ETV1 and ERG responses. In a complementary way, repression of MET led to decrease ETV1/ERG‐induced cell migration and invasion capacities. Taken together, these results depicted that MET and ETV1/ERG act together to contribute to a more aggressive behaviour in an interplaying regulation activity and this effect can be retrieve in the two hormone‐independent models PC3M and PC3, reinforcing our arguments.

To elucidate this interplay, we next focalised on the PC3M model to explore the relevance of these data with regard to the target genes involved and *in vivo* in mice, preferably because PC3M model presents more aggressive phenotypes than PC3.

We thus performed a comparative transcriptomic analysis for control or ETV1/ERG overexpressing cells, in condition of MET activation or not through HGF stimulation.

The analysis allows us to explore different levels of information. Firstly, the genes regulated by ETV1, those regulated by ERG and a comparison to identify common profiles. Secondly, the genes regulated by MET. And finally, the combination of the two, which is the more interesting for deciphering MET/ETV1‐ERG interplay.

Comparison of the ETV1 and ERG programs showed that they share a large number of regulated genes, the number being smoothed by the much smaller number of regulated genes in ETV1 compared with ERG. This difference is most likely due to the higher level of endogenous ETV1 expression compared to none for ERG (as observed in RT‐qPCR and transcriptomic analysis), automatically reducing the number of up‐ or down‐regulated genes for ETV1.

Over and above the number of genes, ETV1 and ERG share the same expression profiles in terms of classification via PANTHER 18.0, and in particular in the “cellular process” and “biological regulation” categories and their corresponding sub‐categories. These sub‐categories included biological and metabolic processes commonly observed in transcriptomic analyses of cancer cells, such as cell cycle regulation, motility, adhesion, communication, developmental process, signal transduction or stimulus response. This is consistent with the effects induced by ETV1 and ERG on phenotypic assays in PC3M cells. The same conclusion can be drawn for the HGF stimulation condition, showing that MET activation induces a transcriptomic profile that overlaps with those of ETV1 and ERG.

Thanks to the classification according to the gene expression folds, it appears that numerous genes (128) are regulated in the same way by ETV1 and ERG factors as well as HGF stimulation, with some of them highly up‐ or down‐regulated. Among them, we could find genes or family of genes well known for their implication in cellular and biological processes inherent to migration, invasion and metastasis. This is the case for genes in the IL, PCDH, ROBO, SEMA, TGFβ, TNF or FGF families represented by *IL11*, *IL13RA2*, *IL1RN*, *IL20RB*, *IL7R*, *PCDH1*, *ROBO4*, *TGFB2*, *TNFAIP3* or *FG14*. These genes or gene families are of particular interest as they belong to gene ontology pathways such as migration, invasion, osteoclast differentiation or bone metastasis and may be very interesting to explore [55, 56, 57].

These results underline that MET activation induced by its ligand HGF induces a gene expression profile common to ETV1 and ERG, thereby corroborating the phenotypic results obtained with cellular models. With the aim of identifying the target genes for ETV1/ERG/MET cooperation, we were able to define a category of “synergistic gene regulation” comprising five genes that are commonly up‐ or down‐regulated to a significant degree by ETV1 and ERG and for which this regulation is amplified by HGF stimulation in the ETV1 + HGF and ERG+HGF conditions. These genes, *AREG*, *CXCR4*, *IL11*, *KITLG* and *OSCAR*, are known to play a significant role in tumour progression and metastasis promotion of prostate cancer as well as colorectal or pancreatic cancers [58, 59, 60]. *AREG* and *OSCAR*, which are among the highly up‐regulated genes, are of particular interest. *AREG* an epidermal growth factor, is implicated in diverse biological processes, including angiogenesis and metastasis formation [60, 61, 62, 63]. *OSCAR* is linked to osteoclast differentiation and the formation of metastases, making it a promising target for bone metastasis [64, 65, 66]. Quite obviously, these fives genes could be interesting candidates for a molecular signature in prostate cancer. Nevertheless, further studies are needed to define their central role in the collaborative interaction between ETV1/ERG transcription factors and the MET receptor, but for now our results show the ETV1/ERG/MET cooperation in the expression of interesting genes involved in cancer progression.

Finally, we performed *in vivo* experiment with ETV1/ERG overexpressing or control PC3M cells and used the Capmatinib, a tyrosine kinase inhibitor targeting MET, to explore the importance of the MET/ETV1/ERG relationship. For that, we used a model of HGF humanised mice (hHGFki) that has a knock‐in of human HGF permitting to activate the human MET at the surface of the injected human tumour cells. In fact, murine HGF cannot activate human MET unlike human HGF, able to activate the murine MET receptor. Subcutaneous injection of cells from the PC3M model demonstrated that overexpression of ETV1 and ERG induced greater tumour growth than control cells throughout the evaluation process.

Treatment with Capmatinib administered to mice during experimentation reduced the tumour growth capacity of PC3M cells overexpressing ETV1 as ERG, whereas the effect was not significant on control cells. These results corroborate those obtained *in vitro* and demonstrate the importance of the interrelationship between the ETV1 and ERG factors and the MET signalling pathway in the ability of cells to develop their tumour‐like properties. Furthermore, the *in vivo* results, using a model in which HGF is produced constitutively, are more pronounced than in cells (increase in MET expression visible in IHC which could also be accompanied by an increase in phosphorylation of the receptor) and are in favour of a MET‐ETV1/ERG cooperation probably involving a convergence of their activity on biological responses rather than on activation loops, even though the latter could contribute. Capmatinib has been approved by FDA in 2020 to be clinically used for metastatic non‐small cell lung cancer. Currently tested in more than forty clinical trials in different cancers, Capmatinib is one of the tyrosine kinase inhibitor treatments to be considered. These results suggest that Capmatinib is a promising treatment option for MET‐ and ETV1/ERG‐expressing prostate cancer patients, with potentially greater sensitivity to TKIs in patients with ETV1 or ERG co‐expression. Indeed, this comparative study shows that, in our cellular models of prostate cancer, there is no significant difference in terms of functionality, regulatory pathways or interface with MET between ETV1 and ERG, which would suggest a common role in prostate cancer, the specificity being rather linked to the fact that the tumours present one or other of the gene fusions, knowing that their presence is mutually exclusive [67]. Of course, all this remains to be verified on samples of tumours from patients at advanced stages of the disease.

## 5 Conclusions

**Fig. 9:**
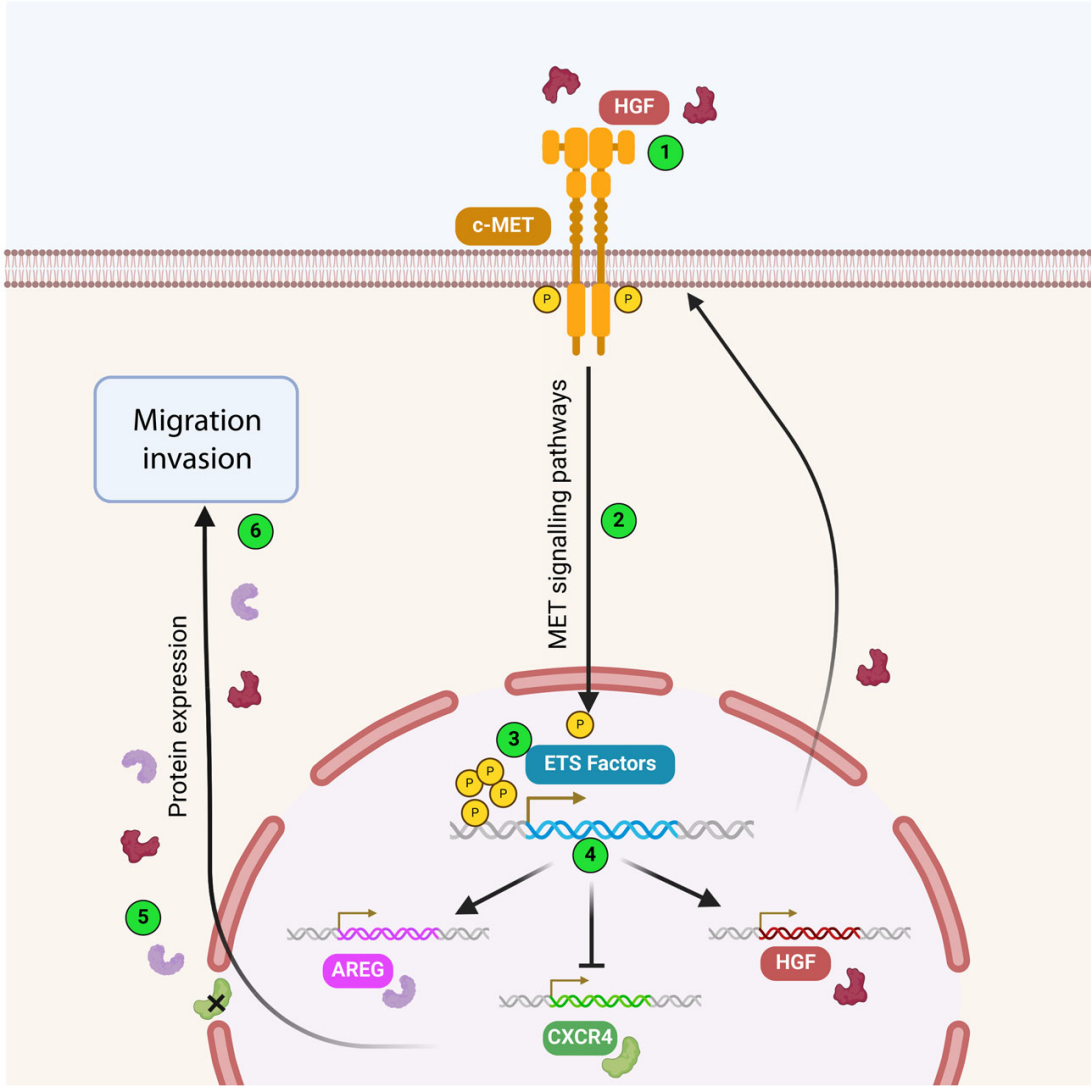
Schematic representation of the potential regulation loop of MET and ETS collaboration. (1) MET is activated by HGF leading to its phosphorylation and (2) the activation of its downstream pathways. (3) ETS factors are activated potentially through MET pathway. (4) ETS target genes are up‐ or down‐regulated (5) leading to particular protein expression and a potential loop thanks to MET and HGF up‐regulation. (6) The activation loop led to the migration and invasion of prostate cancer cells. Created with Biorender.com.

Our study highlights, for the first time in prostate cancer, the impact of collaboration between ETV1/ERG and the MET receptor via a potential self‐regulation, illustrated in Fig. 9. Together, they are responsible for a more aggressive tumour phenotype in prostate cells, promoting prostate cancer progression in mice. In this report, we identify five commonly regulated target genes that could become a potential molecular signature for the progression of prostate cancer to advanced stages. This knowledge provides a new basis for the development of MET‐targeted therapies, which could interfere with MET signalling pathways and, consequently, with ETS transcription factors, thereby reducing progression to the aggressive stages of prostate cancer.

## Conflict of interest

The authors declare to have no conflict of interest.

## Author contributions

EC, DT and ACL conceived and planned the experiments. EC and ACL designed and created the cellular models. EC, CB, NV, AV and ACL performed *in vitro* experiments and processed the data. MDC contributed to the ERG cell model and immunofluorescence planning. ACL and EC conceived the design of animal experiments and processed the experimental data and analysis of the results. MJT helped to animal experiments. EC, CB, MF, SS and ACL contributed to transcriptomic analysis. EC and ACL wrote the manuscript with input from all authors. ACL and DT contributed to funding acquisition and ACL directed the project.

### Peer review

The peer review history for this article is available at https://www.webofscience.com/api/gateway/wos/peer‐review/10.1002/1878‐0261.13739.

## Supporting information


**Fig. S1.** Analysis of MET expression and signalling pathway activation in PC3 cells.
**Fig. S2.** Analysis of ETV1, ERG, MET and HGF expression in established PC3 cells overexpressing ETV1 and ERG.
**Fig. S3.** Measurement of the HGF secretion capacities of PC3M and PC3 cells.
**Fig. S4.** Migration and invasion capacities of ETV1 and ERG overexpressing PC3 cells treated or not by HGF or after MET silencing.
**Fig. S5.** Measurement of the proliferation capacities of PC3M and PC3 cells.
**Fig. S6.** MET expression and signalling pathway activation inhibited by Capmatinib.
**Fig. S7.** Migration capacities of ETV1 and ERG overexpressing PC3M cells treated or not by HGF and Capmatinib.
**Fig. S8.** phosphoMET expression by immunochemistry in control, ETV1 and ERG tumours.
**Fig. S9.** PCNA expression by immunochemistry in control, ETV1 and ERG tumours.
**Fig. S10.** “Biological regulation” charts of ERG, ETV1 and HGF‐stimulated PC3M conditions.

**Table S1.** Oligonucleotide sequences.
**Table S2.** List of known target genes described in the literature for ERG, ETV1 and MET found in the transcriptomic analysis.

**Table S3.** Excel file presenting the transcriptomic analysis group classification of the “cellular process” and “biological regulation” sub‐categories.

## Data Availability

Sequencing data have been submitted to the SRA database from NCBI (https://www.ncbi.nlm.nih.gov/sra). They are accessible under the PRJNA1032721 BioProject number Id. The nucleotide sequence data that support the findings in this study are openly available in the European Nucleotide Archive (ENA) at EMBL‐EBI at https://www.ebi.ac.uk/ena/browser/view/ [PRJEB67887], accession number [PRJEB67887].
