# Supplementary material for: Functional interaction between receptor tyrosine kinase MET and ETS transcription factors promotes prostate cancer progression
Source: Mol Oncol. 2024 Oct 7;19(2):474–95. doi: 10.1002/1878-0261.13739 (PMC11793009; doi:10.1002/1878-0261.13739)
Supplement: Supplementary file 1 — Fig. S1. Analysis of MET expression and signalling pathway activation in PC3 cells. Fig. S2. Analysis of ETV1, ERG, MET and HGF expression in established PC3 cells overexpressing ETV1 and ERG. Fig. S3. Measurement of the HGF secretion capacities of PC3M and PC3 cells. Fig. S4. Migration and invasion capacities of ETV1 and ERG overexpressing PC3 cells treated or not by HGF or after MET silencing. Fig. S5. Measurement of the proliferation capacities of PC3M and PC3 cells. Fig. S6. MET expression and signalling pathway activation inhibited by Capmatinib. Fig. S7. Migration capacities of ETV1 and ERG overexpressing PC3M cells treated or not by HGF and Capmatinib. Fig. S8. phosphoMET expression by immunochemistry in control, ETV1 and ERG tumours. Fig. S9. PCNA expression by immunochemistry in control, ETV1 and ERG tumours. Fig. S10. “Biological regulation” charts of ERG, ETV1 and HGF‐stimulated PC3M conditions. [file MOL2-19-474-s003.zip › Figures_Supplementary.pdf]

FIGURE S1

(A)

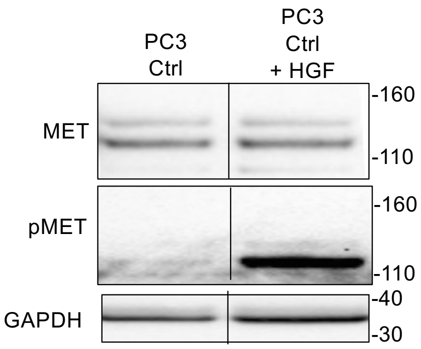
PC3
Ctrl
PC3
Ctrl
+ HGF
MET
pMET
GAPDH
-160
-110
-160
-110
-40
-30

(B)

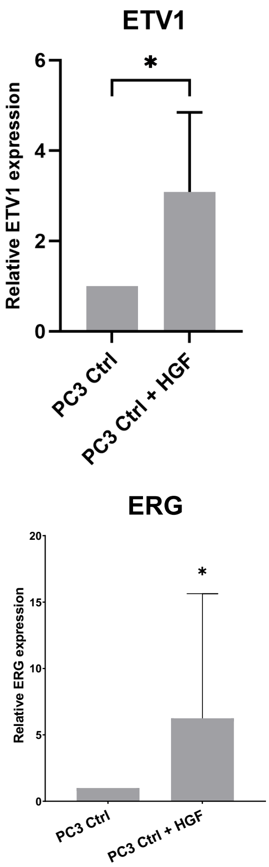
ETV1
Relative ETV1 expression
6
4
2
0
*
PC3 Ctrl
PC3 Ctrl + HGF
ERG
Relative ERG expression
20
15
10
5
0
*
PC3 Ctrl
PC3 Ctrl + HGF

FIGURE S2

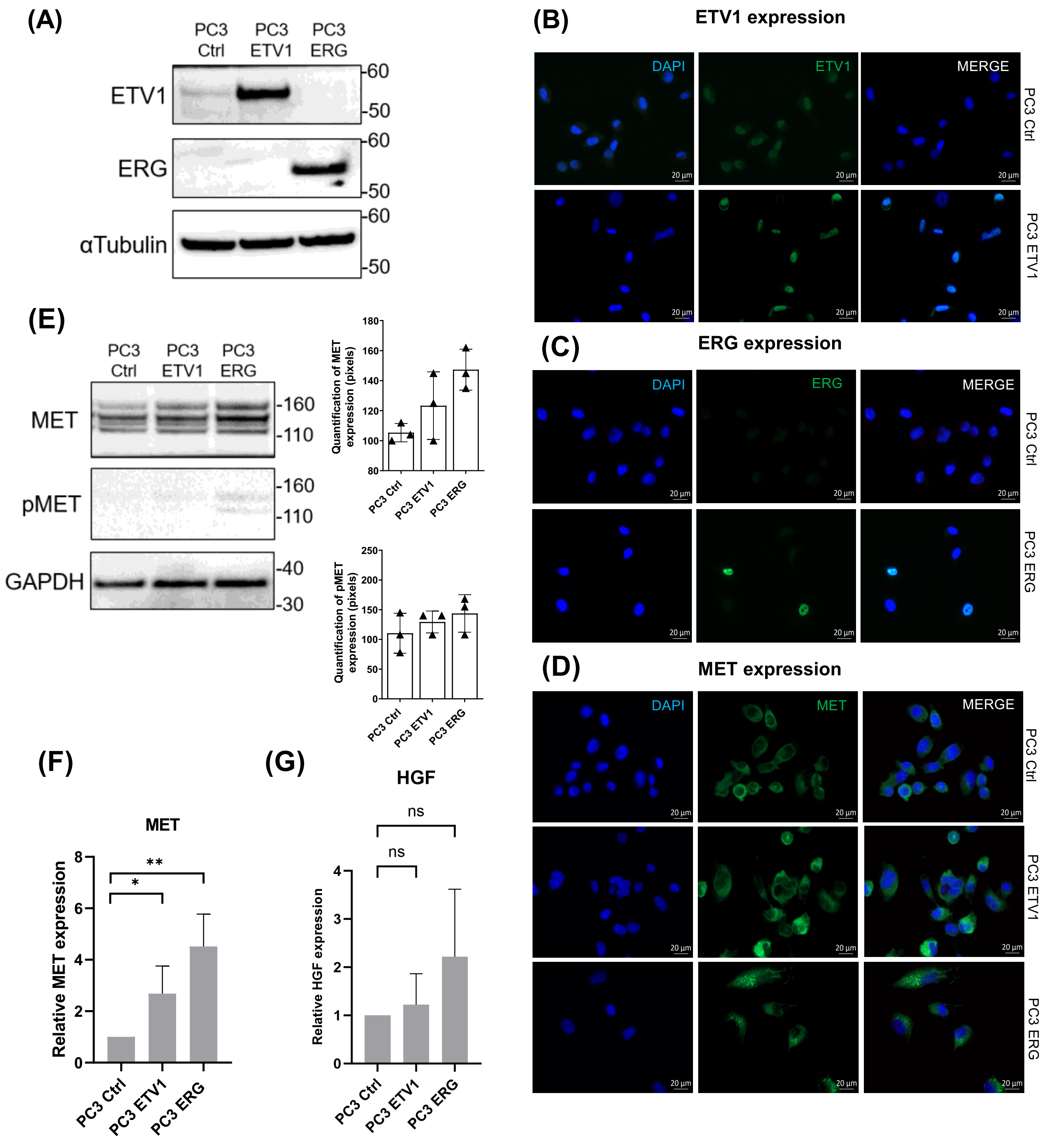
(A)
PC3 Ctrl
PC3 ETV1
PC3 ERG
ETV1
ERG
αTubulin
-60
-50
(B)
ETV1 expression
DAPI
ETV1
MERGE
PC3 Ctrl
PC3 ETV1
20 µm
(C)
ERG expression
DAPI
ERG
MERGE
PC3 Ctrl
PC3 ERG
(D)
MET expression
DAPI
MET
MERGE
PC3 Ctrl
PC3 ETV1
PC3 ERG
(E)
MET
pMET
GAPDH
-160
-110
-40
-30
Quantification of MET expression (pixels)
Quantification of pMET expression (pixels)
(F)
MET
Relative MET expression
*
**
(G)
HGF
ns
Relative HGF expression

FIGURE S3

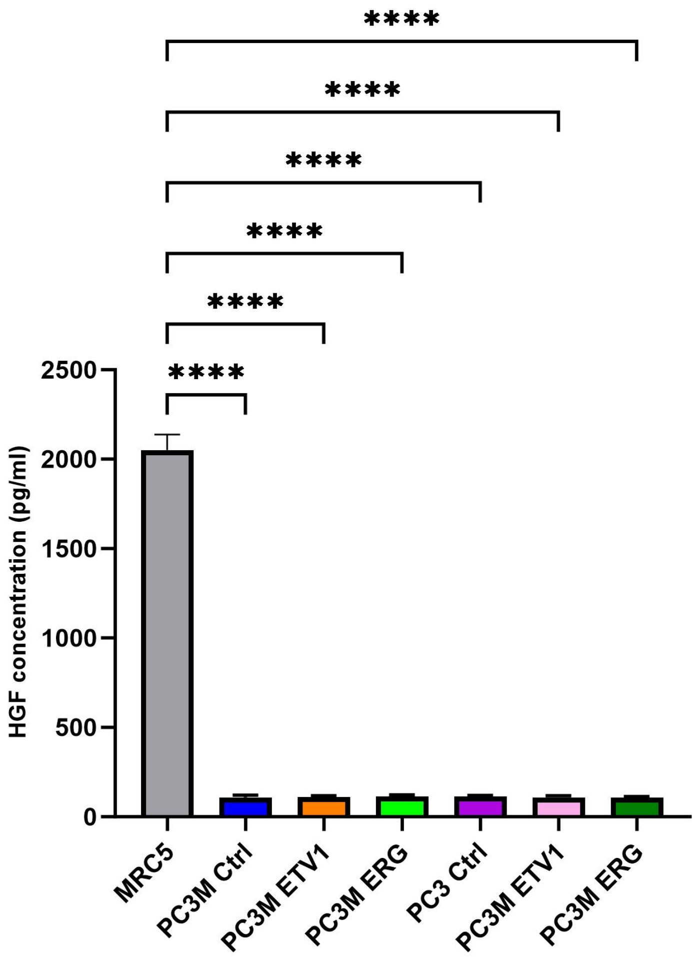

****
****
****
****
****
****
HGF concentration (pg/ml)
2500
2000
1500
1000
500
0
MRC5
PC3M Ctrl
PC3M ETV1
PC3M ERG
PC3 Ctrl
PC3M ETV1
PC3M ERG

FIGURE S4

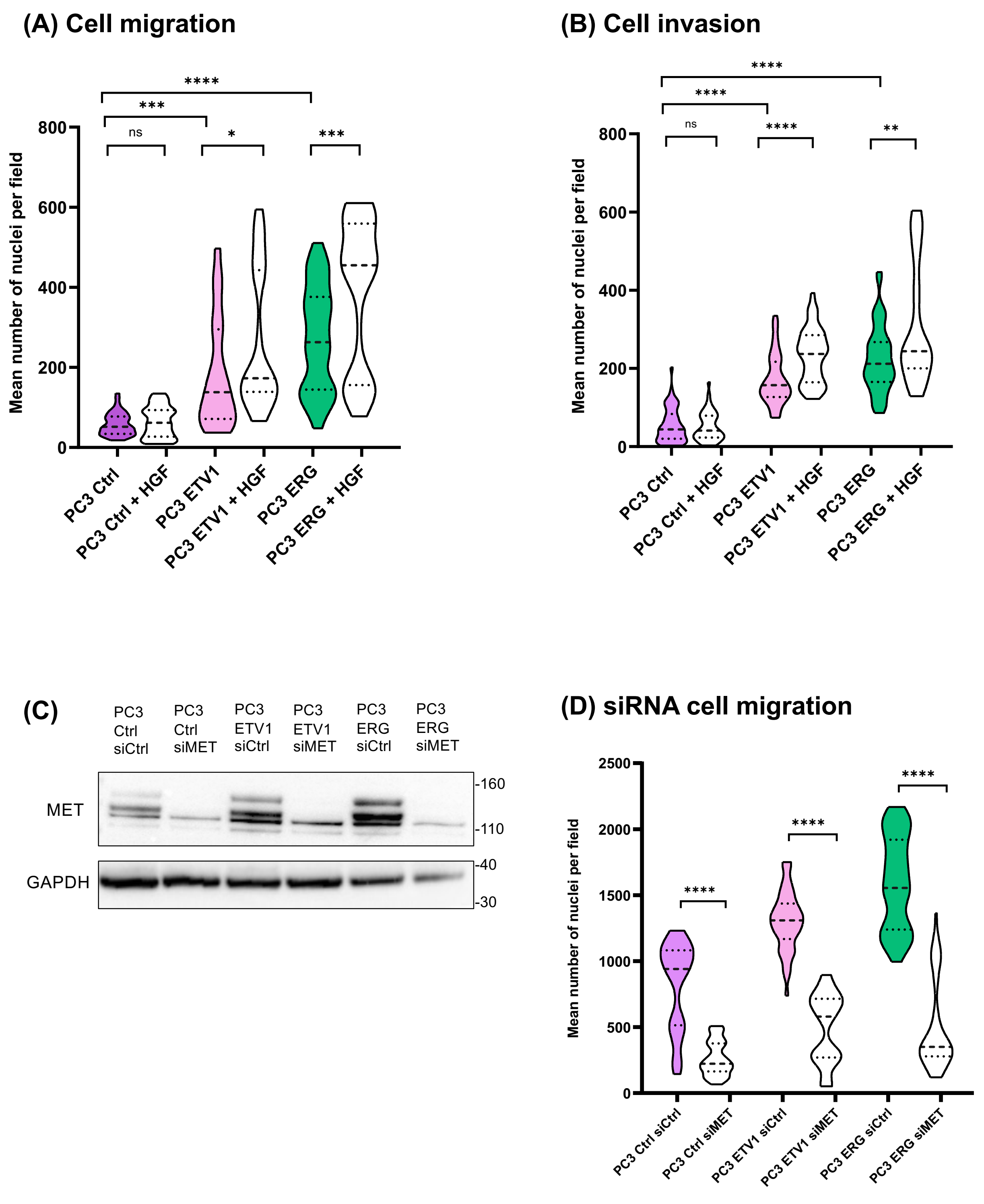

(A) Cell migration
(B) Cell invasion
Mean number of nuclei per field
PC3 Ctrl
PC3 Ctrl + HGF
PC3 ETV1
PC3 ETV1 + HGF
PC3 ERG
PC3 ERG + HGF
(C)
PC3 Ctrl siCtrl
PC3 Ctrl siMET
PC3 ETV1 siCtrl
PC3 ETV1 siMET
PC3 ERG siCtrl
PC3 ERG siMET
MET
GAPDH
-160
-110
-40
-30
(D) siRNA cell migration

FIGURE S5

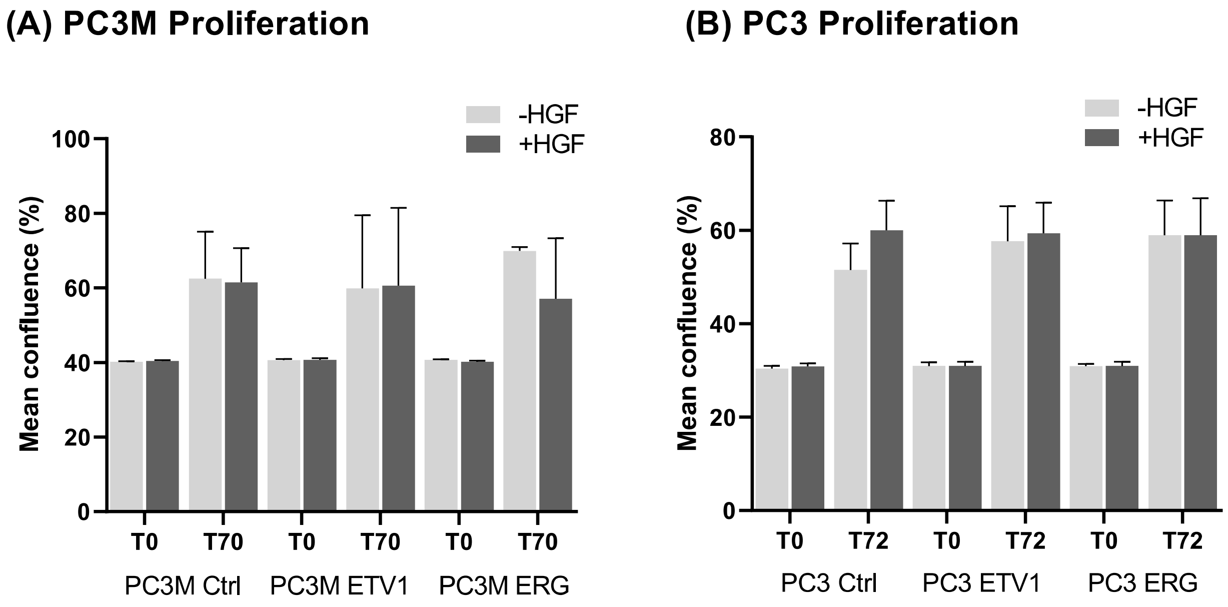
(A) PC3M Proliferation
-HGF
+HGF
Mean confluence (%)
100
80
60
40
20
0
T0
T70
T0
T70
T0
T70
PC3M Ctrl
PC3M ETV1
PC3M ERG
(B) PC3 Proliferation
-HGF
+HGF
Mean confluence (%)
80
60
40
20
0
T0
T72
T0
T72
T0
T72
PC3 Ctrl
PC3 ETV1
PC3 ERG

FIGURE S6

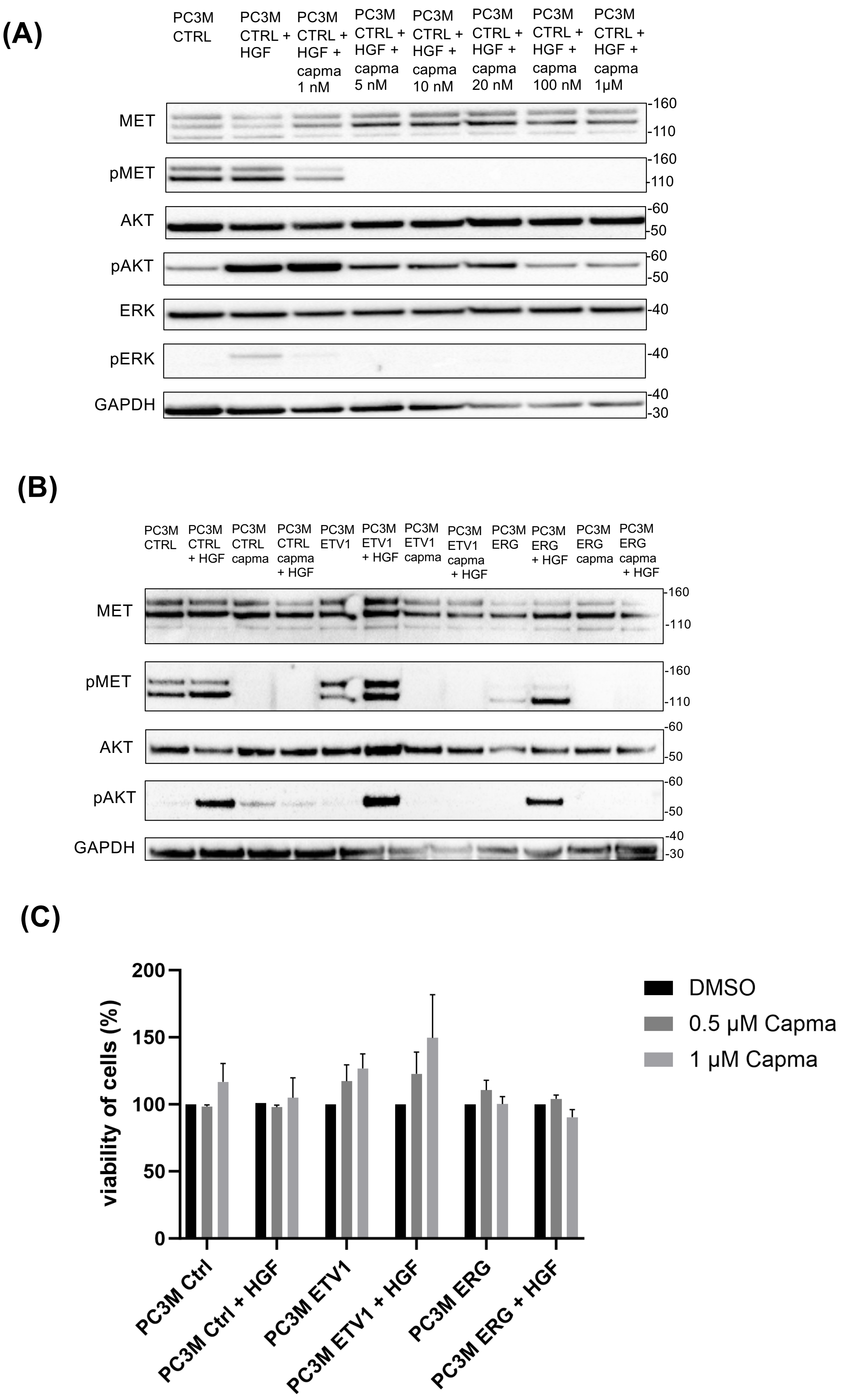

(A)
PC3M CTRL
PC3M CTRL + HGF
PC3M CTRL + HGF + capma 1 nM
PC3M CTRL + HGF + capma 5 nM
PC3M CTRL + HGF + capma 10 nM
PC3M CTRL + HGF + capma 20 nM
PC3M CTRL + HGF + capma 100 nM
PC3M CTRL + HGF + capma 1μM
MET
pMET
AKT
pAKT
ERK
pERK
GAPDH
-160
-110
-60
-50
-40
-30
(B)
PC3M CTRL
PC3M CTRL + HGF
PC3M CTRL capma
PC3M CTRL capma + HGF
PC3M ETV1
PC3M ETV1 + HGF
PC3M ETV1 capma
PC3M ETV1 capma + HGF
PC3M ERG
PC3M ERG + HGF
PC3M ERG capma
PC3M ERG capma + HGF
MET
pMET
AKT
pAKT
GAPDH
(C)
viability of cells (%)
200
150
100
50
0
DMSO
0.5 μM Capma
1 μM Capma
PC3M Ctrl
PC3M Ctrl + HGF
PC3M ETV1
PC3M ETV1 + HGF
PC3M ERG
PC3M ERG + HGF

FIGURE S7

Cell migration

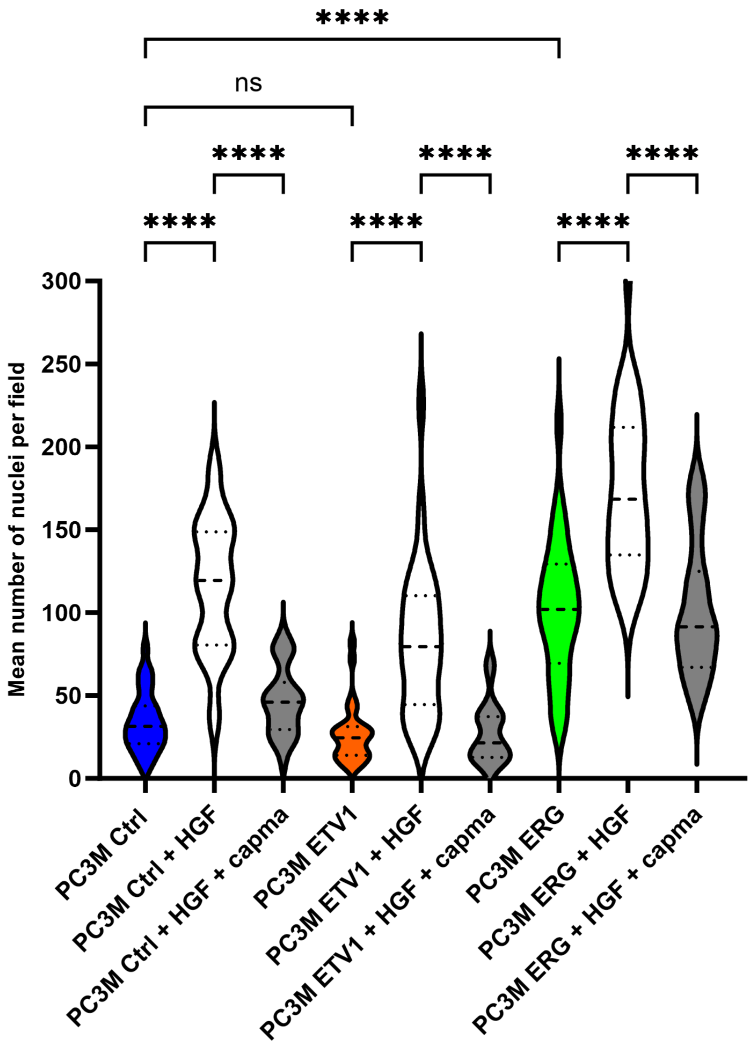

FIGURE S8

(A)

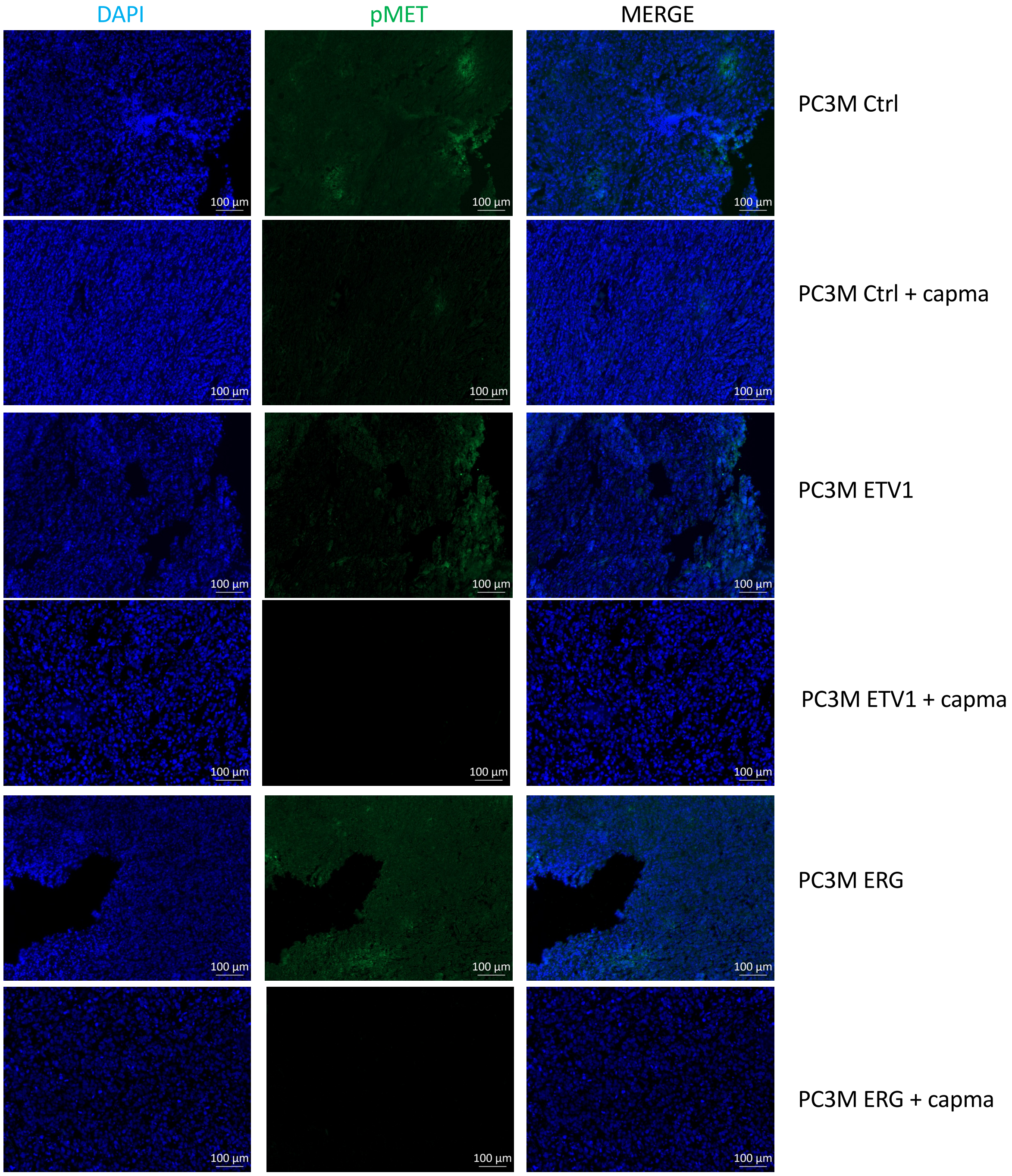
DAPI
pMET
MERGE
PC3M Ctrl
PC3M Ctrl + capma
PC3M ETV1
PC3M ETV1 + capma
PC3M ERG
PC3M ERG + capma
100 µm

(B)

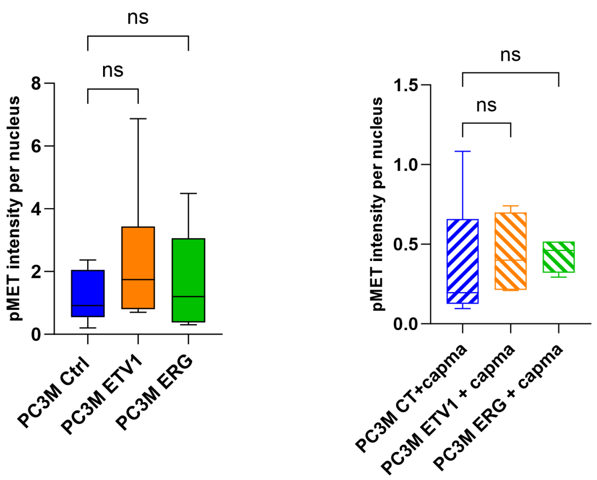
ns
ns
pMET intensity per nucleus
0
2
4
6
8
PC3M Ctrl
PC3M ETV1
PC3M ERG
ns
ns
pMET intensity per nucleus
0.0
0.5
1.0
1.5
PC3M CT+capma
PC3M ETV1 + capma
PC3M ERG + capma

FIGURE S9

(A)

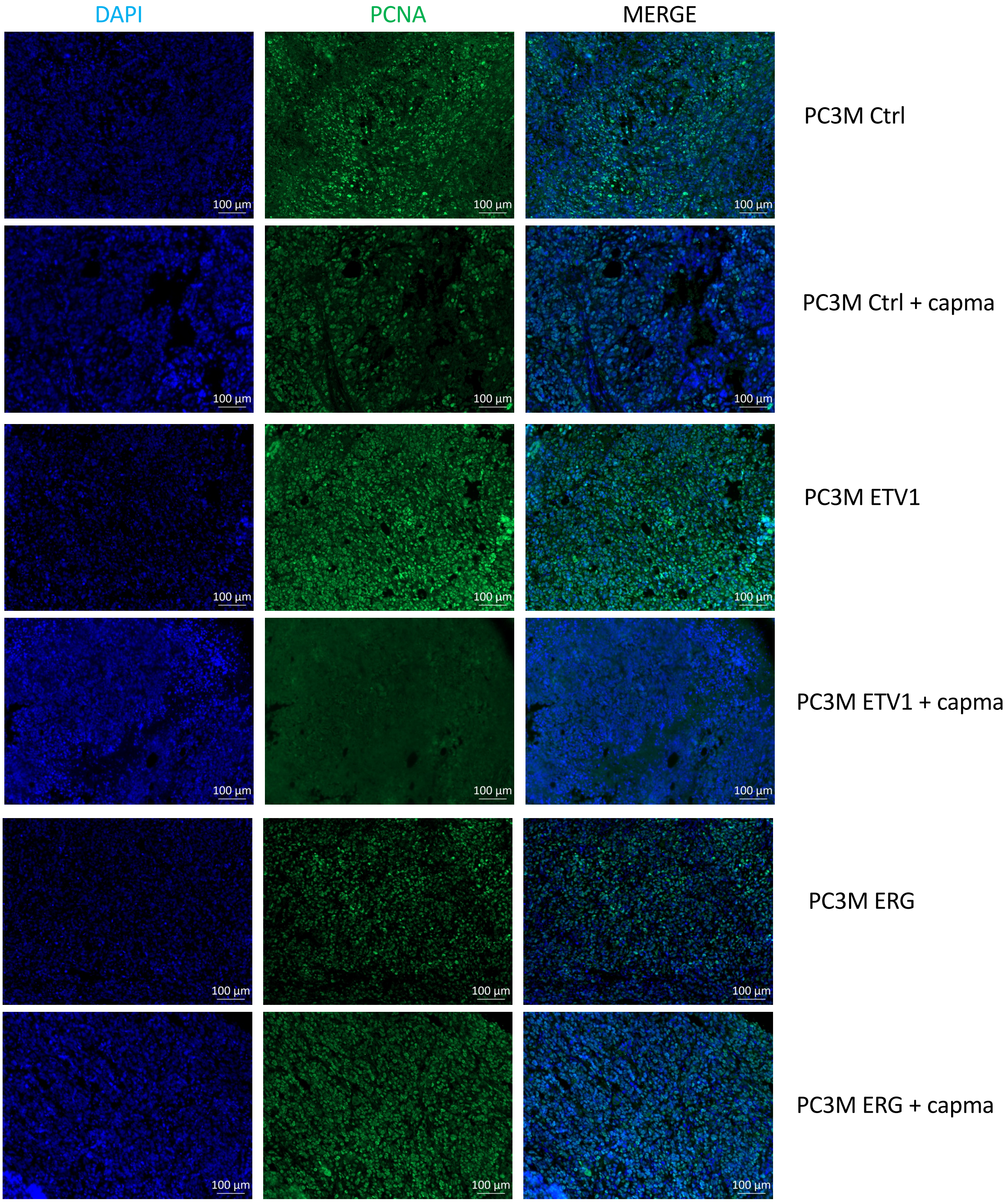
DAPI
PCNA
MERGE
100 µm
PC3M Ctrl
PC3M Ctrl + capma
PC3M ETV1
PC3M ETV1 + capma
PC3M ERG
PC3M ERG + capma

(B)

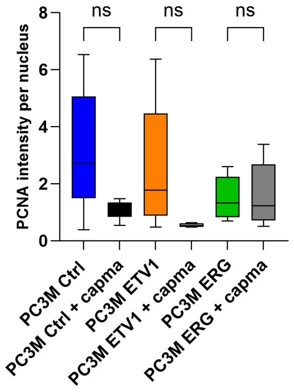
PCNA intensity per nucleus
0
2
4
6
8
ns
ns
ns
PC3M Ctrl
PC3M Ctrl + capma
PC3M ETV1
PC3M ETV1 + capma
PC3M ERG
PC3M ERG + capma

FIGURE S10

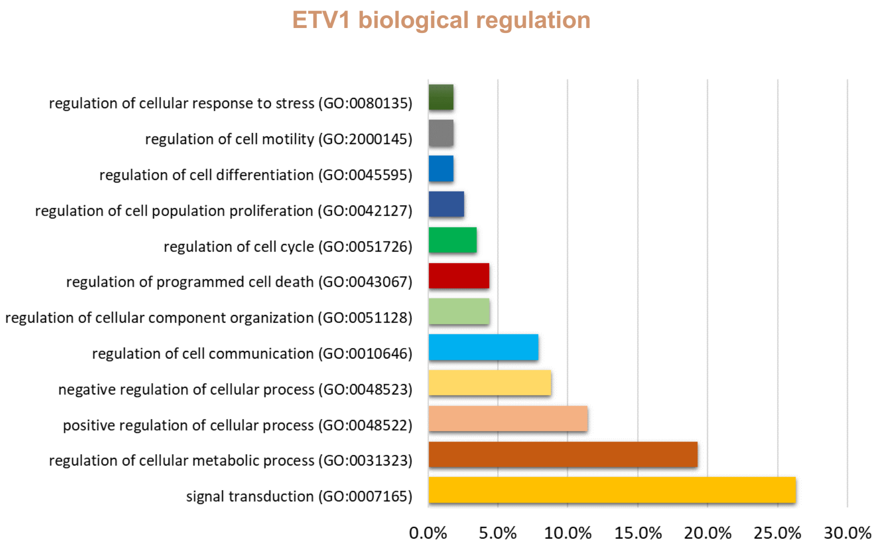
ETV1 biological regulation
regulation of cellular response to stress (GO:0080135)
regulation of cell motility (GO:2000145)
regulation of cell differentiation (GO:0045595)
regulation of cell population proliferation (GO:0042127)
regulation of cell cycle (GO:0051726)
regulation of programmed cell death (GO:0043067)
regulation of cellular component organization (GO:0051128)
regulation of cell communication (GO:0010646)
negative regulation of cellular process (GO:0048523)
positive regulation of cellular process (GO:0048522)
regulation of cellular metabolic process (GO:0031323)
signal transduction (GO:0007165)
0.0% 5.0% 10.0% 15.0% 20.0% 25.0% 30.0%

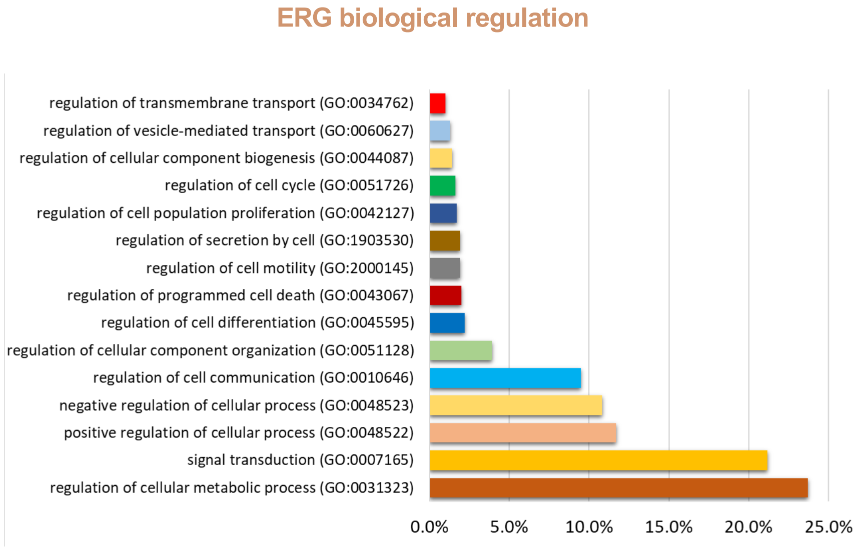
ERG biological regulation
regulation of transmembrane transport (GO:0034762)
regulation of vesicle-mediated transport (GO:0060627)
regulation of cellular component biogenesis (GO:0044087)
regulation of cell cycle (GO:0051726)
regulation of cell population proliferation (GO:0042127)
regulation of secretion by cell (GO:1903530)
regulation of cell motility (GO:2000145)
regulation of programmed cell death (GO:0043067)
regulation of cell differentiation (GO:0045595)
regulation of cellular component organization (GO:0051128)
regulation of cell communication (GO:0010646)
negative regulation of cellular process (GO:0048523)
positive regulation of cellular process (GO:0048522)
signal transduction (GO:0007165)
regulation of cellular metabolic process (GO:0031323)
0.0% 5.0% 10.0% 15.0% 20.0% 25.0%

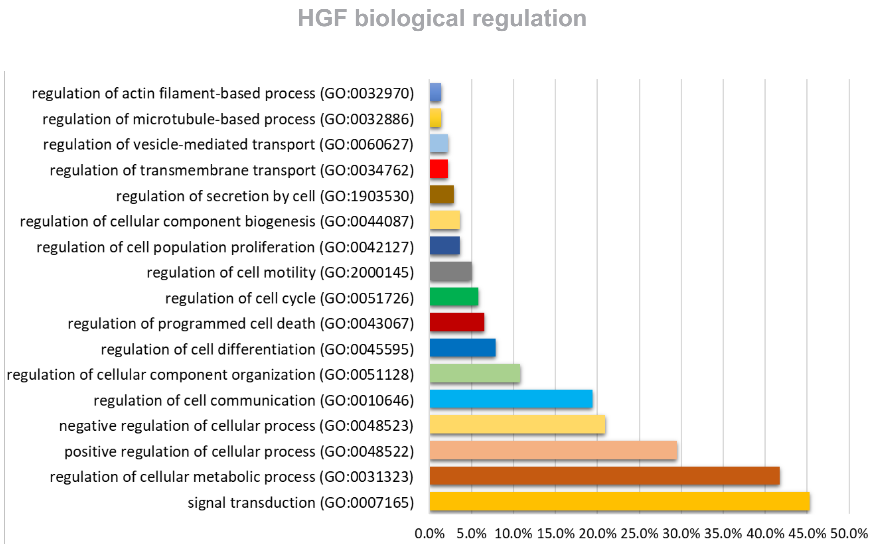
HGF biological regulation
regulation of actin filament-based process (GO:0032970)
regulation of microtubule-based process (GO:0032886)
regulation of vesicle-mediated transport (GO:0060627)
regulation of transmembrane transport (GO:0034762)
regulation of secretion by cell (GO:1903530)
regulation of cellular component biogenesis (GO:0044087)
regulation of cell population proliferation (GO:0042127)
regulation of cell motility (GO:2000145)
regulation of cell cycle (GO:0051726)
regulation of programmed cell death (GO:0043067)
regulation of cell differentiation (GO:0045595)
regulation of cellular component organization (GO:0051128)
regulation of cell communication (GO:0010646)
negative regulation of cellular process (GO:0048523)
positive regulation of cellular process (GO:0048522)
regulation of cellular metabolic process (GO:0031323)
signal transduction (GO:0007165)
0.0% 5.0% 10.0% 15.0% 20.0% 25.0% 30.0% 35.0% 40.0% 45.0% 50.0%
