# Supplementary material for: Functional interaction between receptor tyrosine kinase MET and ETS transcription factors promotes prostate cancer progression
Source: Mol Oncol. 2024 Oct 7;19(2):474–95. doi: 10.1002/1878-0261.13739 (PMC11793009; doi:10.1002/1878-0261.13739)
Supplement: Supplementary file 1 — Fig. S1. Analysis of MET expression and signalling pathway activation in PC3 cells. Fig. S2. Analysis of ETV1, ERG, MET and HGF expression in established PC3 cells overexpressing ETV1 and ERG. Fig. S3. Measurement of the HGF secretion capacities of PC3M and PC3 cells. Fig. S4. Migration and invasion capacities of ETV1 and ERG overexpressing PC3 cells treated or not by HGF or after MET silencing. Fig. S5. Measurement of the proliferation capacities of PC3M and PC3 cells. Fig. S6. MET expression and signalling pathway activation inhibited by Capmatinib. Fig. S7. Migration capacities of ETV1 and ERG overexpressing PC3M cells treated or not by HGF and Capmatinib. Fig. S8. phosphoMET expression by immunochemistry in control, ETV1 and ERG tumours. Fig. S9. PCNA expression by immunochemistry in control, ETV1 and ERG tumours. Fig. S10. “Biological regulation” charts of ERG, ETV1 and HGF‐stimulated PC3M conditions. [file MOL2-19-474-s003.zip › Legends_Supplementary.docx]

**Supplementary legends**

**Figure S1. Analysis of MET expression and signalling pathway activation in PC3 cells.** (A) Protein expression of MET, pMET, AKT, pAKT, ERK and pERK was analysed by Western Blot in PC3 Ctrl cell lines stimulated or not by HGF during 30 minutes. The membrane was first probed with antibodies directed against pMET, pAKT, pERK and reprobed sequentially using an anti- MET, AKT and ERK antibodies on each corresponding membrane. Expression was compared with the reference protein GAPDH. (B). The transcriptional expression of ETV1 and ERG was analysed by RT-qPCR in PC3 Ctrl stimulated or not by HGF during 24h. Expression was normalised to the expression of the reference gene *TBP* (*n=3*). Data are means of 3 experiments analysed by Two-way ANOVA -/+ SD. ns : non-significant, * : p < 0,05.

**Figure S2. Analysis of ETV1, ERG, MET and HGF expression in established PC3 cells overexpressing ETV1 and ERG.** (A). Protein expression of ETV1 and ERG was analysed by Western Blot in PC3 Ctrl, PC3 ETV1 (upper panel) and PC3 ERG cell lines (middle panel). Expression was compared with the reference protein alpha Tubulin (lower panel). (B)(C)(D). Protein expression of ETV1 (B), ERG (C) and MET (D) was detected by immunofluorescence in PC3 Ctrl, PC3 ETV1 and PC3 ERG cell lines. Expression was compared with nuclear staining using DAPI. The merge signal was illustrated in the panels of the right. (E). Protein expression of MET (upper panel) and pMET (middle panel) was analysed by Western Blot in PC3 Ctrl, ETV1 and ERG cell lines. The membrane was first probed with anti- pMET antibody and reprobed sequentially using an anti- MET antibody. Expression was quantified (*n=3*) and compared with the reference protein GAPDH (lower panel). (F)(G) Transcriptional expression of *MET* (F) and *HGF* (G) was analysed by RT-qPCR in PC3 Ctrl, PC3 ETV1 and PC3 ERG cell lines. Expression was normalised to the expression of the *TBP* reference gene (*n=3*). Data are means of 3 experiments analysed by One-way ANOVA for Western blot and Two-way ANOVA for RT-qPCR -/+ SD. ns : non-significant, * : p < 0,05 ; ** : p < 0,005.

**Figure S3. Measurement of the HGF secretion capacities of PC3M and PC3 cells.** ELISA tests were performed with the Human HGF instant ELISA kit, Invitrogen, using extracts of cell supernatants and expressed as pg/ml HGF concentration. Data are analysed by One-way ANOVA -/+ SD. **** : p < 0,00005.

**Figure S4. Migration and invasion capacities of ETV1 and ERG overexpressing PC3 cells treated or not by HGF or after MET silencing.** (A). Migration tests were carried out in a Boyden chamber by measuring the number of nuclei passing through the membrane after 24 hours for PC3 cells treated by HGF or not (*n=3)* represented by the panel under the graph. (B). Invasion tests were carried out in a Boyden chamber by measuring the number of nuclei passing through the membrane after 24 hours for PC3 cells treated by HGF or not (*n=3)* represented by the panel under the graph. (C). Protein expression of MET was analysed by Western Blot in PC3 Ctrl, ETV1 and ERG cell lines after siRNA treatments (siCtrl and siMET) during 24 hours. For each conditions the same amount of protein was analysed by Western blotting using an antibody directed against MET (upper panel). Expression was compared with the reference protein GAPDH (lower panel). (D). Migration tests were carried out in a Boyden chamber by measuring the number of nuclei passing through the membrane after 24 hours for PC3 cells transfected by siCtrl or siMET during 48h before (*n=3)* represented by the panel under the graph. Data are means of at least 3 experiments analysed by One-way ANOVA -/+ SD. ns : non-significant, * : p < 0,05 ; ** : p < 0,005 ; *** : p < 0,0005 ; **** : p < 0,00005.

**Figure S5. Measurement of the proliferation capacities of PC3M and PC3 cells.** The proliferation tests were carried out with the IncuCyte® by measuring the confluence of the wells as a function of time and represented here at 70 hours for (A) PC3M and at 72 hours for (B) PC3 (*n=3*). Data are means of at least 3 experiments.

**Figure S6. MET expression and signalling pathway activation inhibited by Capmatinib.** (A)(B). The protein expression of MET, pMET, AKT, pAKT, ERK and pERK was analysed by Western Blot in the PC3M cell lines treated or not by Capmatinib during 1h30 and then HGF added during 30 minutes for the receptor activation. The membrane was first probed with antibodies directed against pMET, pAKT, pERK and reprobed sequentially using an anti- MET, AKT and ERK antibodies on each corresponding membrane. Expression was compared with the reference protein GAPDH. (A). Capmatinib dose range from 1nM to 1µM for PC3M control cells. (B). Treatment with 10nM Capmatinib for PC3M Ctrl, PC3M ERG and PC3M ETV1 cells. (C). **Analysis of cell viability of PC3M cell lines by MTT test.** The MTT test was carried out on the different PC3M cells after 24 hours of treatment with 0.5 µM or 1 µM of Capmatinib. The cells were treated with HGF for 24 hours when indicated. The cells were stained with an MTT solution and the absorbance was measured after solubilisation of the cells, the purple colour reflecting the percentage of cell viability (n=3). Data are means of at least 3 experiments.

**Figure S*7*.** **Migration capacities of ETV1 and ERG overexpressing PC3M cells treated or not by HGF and Capmatinib.** Migration tests were carried out in a Boyden chamber by measuring the number of nuclei passing through the membrane after 24 hours for PC3M cells treated by HGF and Capmatinib or not. Data are means of at least 3 experiments analysed by One-way ANOVA -/+ SD. ns : non-significant; **** : p < 0,00005.

**Figure S8. phosphoMET expression by immunochemistry in control, ETV1 and ERG tumours.** (A). MET immunostaining in PC3M Ctrl, PC3M ETV1 and PC3M ERG tumours treated or not with Capmatinib. Expression was compared with nuclear staining using DAPI. The merge signal was illustrated in the panels of the right. (B). Quantification of phosphoMET immunostaining in PC3M Ctrl, PC3M ETV1 and PC3M ERG tumours treated or not with Capmatinib. Quantification was carried out by One-way ANOVA -/+ SD. ns : non-significant.

**Figure S9. PCNA expression by immunochemistry in control, ETV1 and ERG tumours.** (A). MET immunostaining in PC3M Ctrl, PC3M ETV1 and PC3M ERG tumours treated or not with Capmatinib. Expression was compared with nuclear staining using DAPI. The merge signal was illustrated in the panels of the right. (B). Quantification of PCNA immunostaining in PC3M Ctrl, PC3M ETV1 and PC3M ERG tumours treated or not with Capmatinib. Quantification was carried out by One-way ANOVA -/+ SD. ns : non-significant.

**Figure S10. “Biological regulation” charts of ERG, ETV1 and HGF-stimulated PC3M conditions.** Genes were selected by p.value ≥0.05 and fold change ≤1.2 and charted in a graphical representation of their “biological regulation” by PANTHER 18.0 in (A) ERG, (B) ETV1 and (C) HGF compared to PC3M Ctrl.
