# Supplementary material for: Functional interaction between receptor tyrosine kinase MET and ETS transcription factors promotes prostate cancer progression
Source: Mol Oncol. 2024 Oct 7;19(2):474–95. doi: 10.1002/1878-0261.13739 (PMC11793009; doi:10.1002/1878-0261.13739)
Supplement: Supplementary file 2 — Table S1. Oligonucleotide sequences. Table S2. List of known target genes described in the literature for ERG, ETV1 and MET found in the transcriptomic analysis. [file MOL2-19-474-s001.pdf]

Table S1. Oligonucleotide sequences

Gene name	**Oligonucleotide sequences**
Tbp – Forward	5′-TCAAACCCAGAATTGTTCTCCTTAT-3′
Tbp – Reverse	5′-CCTGAATCCCTTTAGAATAGGGTAGA-3′
Etv1 – Forward	5′-CTGAACCCTGTAACTCCTTTCC-3′
Etv1 – Reverse	5′-AGACATCTGGCGTTGGTACATA-3′
Erg – Forward	5′-AACGAGCGCAGAGTTATCGT-3′
Erg – Reverse	5′-GTGAGCCTCTGGAAGTCGTC-3′
Met – Forward	5′-CATGAGCACTGCTTTAATAGG-3′
Met – Reverse	5′-GGACTTCGCTGAATTGACCCATG-3′
Hgf – Forward	5′-AACTCGCAATTGTTTCGTTTT-3′
Hgf – Reverse	5′-GCTCATGGACCCTGGTGCTAC-3′
Il11 – Forward	5′-TCTCTCCTGGCGGACACG-3′
Il11 – Reverse	5′-AATCCAGGTTGTGGTCCCC-3′
Kitlg – Forward	5′GGATGGATGTTTTGCCAAGT-3′
Kitlg – Reverse	5′-TCTTTCACGCATCCACAG-3′
Oscar – Forward	5′-CTGATCCTCCAGCTGCTGAC-3′
Oscar – Reverse	5′-CCAGGCTTGAAAAGTCCAAA-3′
Areg – Forward	5′-GTGGTGCTGTCGCTCTTGATACTC-3′
Areg – Reverse	5′-TCAAATCCATCAGCACTGTGGTC-3′
Cxcr4 – Forward	5′-CAGCAGGTAGCAAAGTGACG-3′
Cxcr4 – Reverse	5′-CAGGGTTCCTTCATGGAGTC-3′

Table S2. List of known target genes described in the literature for ERG, ETV1 and MET found in the transcriptomic analysis

	symbol	geneID	log2FoldChange	padj
**ERG**	MMP1	ENSG00000196611	**0.83893006**	**0.014683114**
	MMP10	ENSG00000166670	**0.149756522**	**0.797720563**
	MMP13	ENSG00000137745	**1.887279858**	**5.23705E-45**
	MMP14	ENSG00000157227	**0.556886873**	**0.055944416**
	MMP16	ENSG00000156103	**0.116821772**	**0.36610226**
	MMP17	ENSG00000198598	**0.452478318**	**0.524121382**
	BMP3	ENSG00000152785	**0.1017727**	**0.405713475**
	BMP4	ENSG00000125378	**0.534630897**	**0.043191358**
	BCL2	ENSG00000171791	**-0.50567338**	**0.020859577**
**ETV1**	MMP1	ENSG00000196611	**0.508392841**	**0.352841532**
	MMP10	ENSG00000166670	**0.284702579**	**0.714073923**
	MMP13	ENSG00000137745	**0.07463208**	**0.859860888**
	BMP1	ENSG00000168487	**-0.4737787**	**0.12384521**
	BMP3	ENSG00000152785	**-0.442180113**	**2.3208E-05**
	BCL2	ENSG00000171791	**-0.05441643**	**0.917446132**
**MET**	MMP1	ENSG00000196611	**0.9991475**	**0.004337899**
	MMP10	ENSG00000166670	**0.707403814**	**0.088729461**
	MMP16	ENSG00000156103	**0.174127047**	**0.153122047**
	MYC	ENSG00000136997	**-0.623549357**	**0.219026939**
	BCL2	ENSG00000171791	**0.262056196**	**0.26778418**
